# 4-Phenylbutyrate restored γ-aminobutyric acid uptake and reduced seizures in *SLC6A1* patient variant-bearing cell and mouse models

**DOI:** 10.1093/braincomms/fcac144

**Published:** 2022-06-06

**Authors:** Gerald Nwosu, Felicia Mermer, Carson Flamm, Sarah Poliquin, Wangzhen Shen, Kathryn Rigsby, Jing Qiong Kang

**Affiliations:** Department of Neurology, Vanderbilt University Medical Center, 465 21st Ave South, Nashville, TN 37232, USA; Neuroscience Graduate Program, Vanderbilt University, Nashville, TN, USA; Vanderbilt Brain Institute, Vanderbilt University, Nashville, TN, USA; Department of Biochemistry, Cancer Biology, Neuroscience and Pharmacology, Meharry Medical College, Nashville, TN, USA; Department of Neurology, Vanderbilt University Medical Center, 465 21st Ave South, Nashville, TN 37232, USA; Vanderbilt Brain Institute, Vanderbilt University, Nashville, TN, USA; Department of Neurology, Vanderbilt University Medical Center, 465 21st Ave South, Nashville, TN 37232, USA; Neuroscience Graduate Program, Vanderbilt University, Nashville, TN, USA; Vanderbilt Brain Institute, Vanderbilt University, Nashville, TN, USA; Department of Neurology, Vanderbilt University Medical Center, 465 21st Ave South, Nashville, TN 37232, USA; Vanderbilt Brain Institute, Vanderbilt University, Nashville, TN, USA; Department of Neurology, Vanderbilt University Medical Center, 465 21st Ave South, Nashville, TN 37232, USA; Neuroscience Graduate Program, Vanderbilt University, Nashville, TN, USA; Vanderbilt Brain Institute, Vanderbilt University, Nashville, TN, USA; Department of Biochemistry, Cancer Biology, Neuroscience and Pharmacology, Meharry Medical College, Nashville, TN, USA; Department of Pharmacology, Vanderbilt University, Nashville, TN, USA; Vanderbilt Kennedy Center of Human Development, Nashville, TN, USA

**Keywords:** GABA transporter 1 (GAT-1), epilepsy, autism, 4-phenylbutyrate acid, chaperone

## Abstract

We have studied the molecular mechanisms of variants in solute carrier Family 6 Member 1 associated with neurodevelopmental disorders, including various epilepsy syndromes, autism and intellectual disability. Based on functional assays of solute carrier Family 6 Member 1 variants, we conclude that partial or complete loss of γ-amino butyric acid uptake due to reduced membrane γ-amino butyric acid transporter 1 trafficking is the primary aetiology. Importantly, we identified common patterns of the mutant γ-amino butyric acid transporter 1 protein trafficking from biogenesis, oligomerization, glycosylation and translocation to the cell membrane across variants in different cell types such as astrocytes and neurons. We hypothesize that therapeutic approaches to facilitate membrane trafficking would increase γ-amino butyric acid transporter 1 protein membrane expression and function. 4-Phenylbutyrate is a Food and Drug Administration-approved drug for paediatric use and is orally bioavailable. 4-Phenylbutyrate shows promise in the treatment of cystic fibrosis. The common cellular mechanisms shared by the mutant γ-amino butyric acid transporter 1 and cystic fibrosis transmembrane conductance regulator led us to hypothesize that 4-phenylbutyrate could be a potential treatment option for solute carrier Family 6 Member 1 mutations. We examined the impact of 4-phenylbutyrate across a library of variants in cell and knockin mouse models. Because γ-amino butyric acid transporter 1 is expressed in both neurons and astrocytes, and γ-amino butyric acid transporter 1 deficiency in astrocytes has been hypothesized to underlie seizure generation, we tested the effect of 4-phenylbutyrate in both neurons and astrocytes with a focus on astrocytes. We demonstrated existence of the mutant γ-amino butyric acid transporter 1 retaining wildtype γ-amino butyric acid transporter 1, suggesting the mutant protein causes aberrant protein oligomerization and trafficking. 4-Phenylbutyrate increased γ-amino butyric acid uptake in both mouse and human astrocytes and neurons bearing the variants. Importantly, 4-phenylbutyrate alone increased γ-amino butyric acid transporter 1 expression and suppressed spike wave discharges in heterozygous knockin mice. Although the mechanisms of action for 4-phenylbutyrate are still unclear, with multiple possibly being involved, it is likely that 4-phenylbutyrate can facilitate the forward trafficking of the wildtype γ-amino butyric acid transporter 1 regardless of rescuing the mutant γ-amino butyric acid transporter 1, thus increasing γ-amino butyric acid uptake. All patients with solute carrier Family 6 Member 1 variants are heterozygous and carry one wildtype allele, suggesting a great opportunity for treatment development leveraging wildtype protein trafficking. The study opens a novel avenue of treatment development for genetic epilepsy via drug repurposing.

## Teaser

The common molecular mechanisms of SLC6A1 variant-mediated neurodevelopmental disorders have been previously identified. The same group now demonstrated that 4-phenylbutyrate, a Food and Drug Administration-approved compound for children with urea cycle disorders, can restore GABA uptake and reduce seizures in patient variant-bearing cell and knockin mouse models.

## Graphical Abstract

**Graphical abstract:**
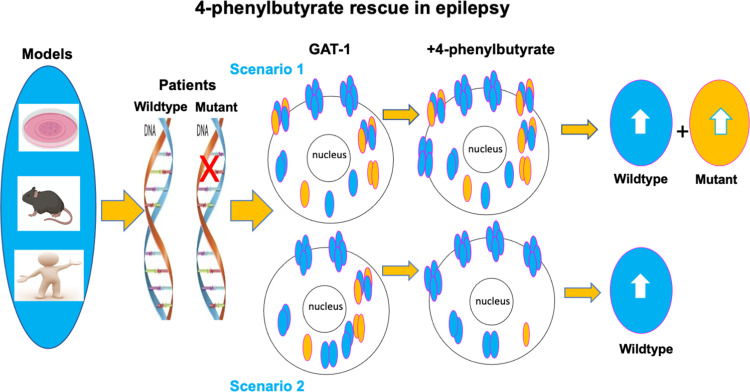

## Introduction

Genetic mutation and the subsequent protein misfolding are a major cause of disease throughout the life span^1–3^ of those affected by solute carrier Family 6 Member 1 (*SLC6A1*) disorders. In childhood, pathogenic variants, or mutations in numerous genes, give rise to a wide range of neurodevelopmental disorders. γ-Aminobutyric acid (GABA) transporter 1 (GAT-1) encoding *SLC6A1* is one of such genes with potential pathogenic variants.^4–6^ *SLC6A1* is a member of the neurotransmitter subgroup of the solute carrier (SLC6) family.^7^ The family includes three GABA transporters (GAT), two glycine transporters and the monoamine transporters such as dopamine, serotonin and norepinephrine. The *SLC6* family transporters are integral membrane proteins characterized by the Na^+^-dependent translocation of small amino acid or amino acid-like substrates. Genetic sequencing has identified that mutations in the family are a major aetiology for neurodevelopmental disorders such as epilepsy and autism.^4,5,8^

We have previously characterized the functional consequences of both missense and nonsense *SLC6A1* mutations^8,9^ and identified the common mechanisms of the molecular pathophysiology underlying the heterogeneous clinical phenotypes. The molecular pathophysiological mechanisms include reduction or loss of GABA uptake, endoplasmic reticulum (ER) retention of the mutant GAT-1 protein and reduced cell surface and total GAT-1 protein expression due to impaired protein trafficking.^8–11^ The mechanisms directly contributing to diminished GAT-1 function include decreased membrane protein trafficking due to protein misfolding and altered protein stability.^8–10^ Our work suggests that the mutant GAT-1 is subject to the common protein surveillance inside the ER as other mutations such as γ-aminobutyric acid type A (GABA_A_) receptor subunit mutations.^12–14^

Our extensive studies in the mutant GABA_A_ receptor and GAT-1 suggest that impaired protein trafficking due to protein misfolding is a common aetiology in genetic epilepsy and neurodevelopmental disorders.^10,11,15,16^ This thus provides a great opportunity for treatment development by leveraging the protein trafficking pathway, via which the misfolded mutant protein is processed. Protein misfolding has been widely studied for later onset neurodegenerative diseases but much less so for early onset childhood disorders such as epilepsy. Our findings on impaired protein trafficking in both GABA_A_ receptors and GAT-1 provide a mechanistic link between genetic epilepsy and neurodegenerative disease. This suggests pharmacological compounds identified for neurodegeneration could be repurposed for neurodevelopmental disorders such as genetic epilepsy. We propose that leveraging the ER pathway and promoting membrane protein trafficking could be a novel treatment target for genetic epilepsy as well as other related disorders.^17^

4-Phenylbutyrate, or 4-phenylbutyric acid (PBA), is a salt of an aromatic fatty acid. It is used to treat urea cycle disorders, as its metabolites offer an alternative pathway to the urea cycle, allowing excretion of excess nitrogen.^18^ PBA is a chaperone that can reduce ER stress^19,20^ while also functioning as a histone deacetylase inhibitor.^21,22^ In this study, we compared the effect of PBA with other chaperones on GABA uptake for eight mutations and evaluated the impact of PBA on the functional rescue in different model systems, including in patient induced pluripotent stem cell (iPSC)-derived astrocytes and knockin mice. Because GAT-1 is expressed in both neurons and astrocytes and because of the unique role of GAT-1 in thalamic astrocytes and the associated seizures phenotypes,^23–26^ we characterized the impact of treatment on GABA uptake activity in astrocytes and neurons derived from patient cells and in two *SLC6A1* mutation knockin mouse models. In knockin mice, we evaluated the effect of PBA by video monitoring synchronized electroencephalogram (EEG) recordings and thus determined the effect of PBA in mitigating seizure activity.

## Materials and methods

### SLC6A1 variant information

The patient variants are selected from the lab cDNA library built based on our previous studies.^5,9,27,28^ Those variants represent both missense and nonsense mutations, exhibit variant locations in both the N- and C-terminus, and display common patterns of functioning and trafficking defects of the variants.

### SLC6A1 mutation knockin mouse models

Both mutations *SLC6A1(A288V)* and *SLC6A1(S295L)*, for which the knockin mouse models have been created, have been extensively characterized in our previous study at the molecular level.^9^ The *SLC6A1(A288V)* mouse line was generated in collaboration with the University of Connecticut health centre, UConn Health and the *SLC6A1(S295L)* mouse line was generated by Shanghai Model Organisms (Shanghai Model Organisms Center, Inc., Cat. No. NM-KI-190014). Both mouse lines are developed with the CRISPR-CAS9 global knockin approach. Both mouse lines are maintained in the C57BL/6J mice (Jax Stock #000664). The mice used for experiments in the study were generated by breeding the heterozygous with the wildtype. Both male and female heterozygous mice were used for breeding for experiments in both mouse lines.

### Cloning of GABA transporter 1

The plasmid cDNA encoding enhanced yellow fluorescent protein (EYFP)-tagged rat GAT-1 has been previously described.^29^ The coding region of rGAT-1 was inserted into pEYFP-C1 (Clontech, Palo Alto, CA, USA). QuikChange site-directed Mutagenesis kit was utilized to introduce the GAT-1 variants into a wildtype GAT-1 plasmid. The product was then amplified via polymerase chain reaction, transformed using DH5α competent cells and plated. A clone was chosen and grown overnight. All the GAT-1 variants were confirmed by DNA sequencing. Both the wildtype and the variant cDNAs were prepared with Qiagen Maxiprep kit.

### Mouse cortical neuron and astrocyte cultures

Mouse cortical neurons were prepared from postnatal Day 0 pups.^9,30^ The neurons used for experiments were at Days 15–17 after culture. Mouse cortical astrocyte cultures were prepared from the postnatal 0- to 3-day-old pups as previously described.^9,17^ Briefly, the cortices of postnatal days 0–3 pups were dissected. The tissues were minced after removing the meninges and then digested with 0.25% trypsin for 10 min at 37°C. The cells were cultured in poly-l-lysine coated 100 mm2 dishes and maintained in Dulbecco’s modified Eagle’s medium supplemented with 10% foetal bovine serum and 1% penicillin/streptomycin. The astrocytes used for experiments were at Passage 2.

### Human patient-derived neurons and astrocytes and transfections in human astrocytes

The patient-derived and CRISPR-corrected iPSCs were obtained in collaboration with Dr Jason Aoto’s laboratory (University of Colorado). The normal human iPSC clone was purchased from Thermo Fisher (A18945). The detailed iPSC culture, solutions and differentiation protocols are described in Supplementary Methods. Briefly, the corrected and patient cell lines were maintained in plates coated with Geltrex (1:50 DMEM/F12) overnight using mTeSR (Stem Cell Technologies). The media were refreshed daily for iPSCs. The differentiation of neural progenitor cells (NPCs) was induced by STEMdiff SMADi neural induction kit from STEM Cell Technologies. The differentiation of inhibitory neurons was based on our previous protocol^9^ (Supplementary Method). The differentiation of astrocytes was initiated using the Astrocyte medium (ScienCell) for ∼30 days, and the cells were passaged at ∼70% confluency.^31^ The differentiation of astrocytes was initiated at NPC Day 5 from Passage 1. The experiments in astrocytes were carried out between 30 and 35 days after differentiation from NPCs. The transfection protocol was the same as in our previous study.^9^

### Radioactive 3H-labelled GABA uptake assay

The radioactive ^3^H-labelled GABA uptake assay in human embryonic kidney (HEK293T) cells, mouse and human astrocytes and neurons have been described in our previous studies^8,9^ and detailed in Supplementary Methods.

### Measurement of surface and total expression of GAT-1 using flow cytometry and confocal microscopy

The protocols used for measurement of surface and total expression of GAT-1 using flow cytometry and confocal microscopy have been described previously for studies on GABA_A_ receptor subunit variants and *SLC6A1* variants.^9,11^ Both the surface and the total GAT-1 protein were probed with rabbit polyclonal anti-rabbit GAT-1, and 10 000 single cells were evaluated. The empty vector pcDNA mock transfected cells and untransfected cells were included as reference each time.

### PBA administration *in vitro* and *in vivo*

For PBA administration in cells, the stocking solution of PBA (2 M) was dissolved in dimethyl sulfoxide (DMSO). A series of concentrations and incubation periods for PBA has been tested and the most optimal dosage (2 mM) and duration (24 h) were identified and used throughout the study. For PBA administration in mice, the dose was chosen based on a dose–response experiment performed using increasing doses of PBA from 100 to 800 mg/kg to determine the most efficacious dose.^32^ Mice of both sexes at 2–4 months of age were dosed with PBA (100 mg/kg, i.p.) or vehicle for 7 days. PBA solution was prepared by dissolving PBA in 0.9% normal saline and then titrating equimolecular amounts of 4-phenylbutyric acid (Sigma, Madrid, Spain) and 1 M potassium hydroxide to pH 7.4. The working solution was stored at 4°C.

### Synchronized video-monitoring EEG recordings

The surgery to implant the EEG head mount and the video-monitoring synchronized EEG recordings were conducted as previously described.^7,8^

### EEG analysis with seizure pro software

For recordings, the sampling rate is set at 400 Hz with a pre-amplifier gain of 100 Hz. EEG and electromyography channels have a filter set at 25 Hz. EEG recordings are scored blindly by a skilled scorer using the Sirenia Seizure Pro software. A power analysis is performed using the theta frequency band of 5–7 Hz. We chose to measure 5–7 Hz spike wave discharges (SWDs) because it is the mouse correlate of 2–4 Hz SWDs in humans.^24,33^ An average power is calculated using baseline recordings and applied to seizure analysis for treatment recordings. Seizures identified by the software were confirmed using video recordings of the period. The Racine scale was used for seizure identification (Stage 1: mouth/facial movements; Stage 2: head nodding; Stage 3: forelimb clonus; Stage 4: rearing; Stage 5: rearing and falling). The identified SWDs were then confirmed with video monitoring and compared across treated and non-treated recordings. The total seizure events over 48 h of EEG recordings were reported.

### Statistical analysis

Data were expressed as mean ± standard error mean. Proteins were quantified by Odyssey software and data were normalized to loading controls and then to the wildtype subunit protein, which was arbitrarily taken as one in each experiment. Fluorescence intensities from confocal microscopy experiments were determined using MetaMorph imaging software, and the measurements were carried out in ImageJ as modified from previous description.^1,7,8,10^ For statistical significance, we used one- or two-way analysis of variance with post hoc Dunnett or Newman–Keuls test. In some cases, unpaired *t*-test or one sample *t*-test was performed (GraphPad Prism, La Jolla, CA, USA), and statistical significance was taken as *P* < 0.05.

### Data availability

The data supporting the findings of this study are available within the article and its Supplementary material.

## Results

### Missense and nonsense SLC6A1 variants caused partial or complete loss of GABA uptake function due to reduced GAT-1 surface protein expression

**Figure 1:**
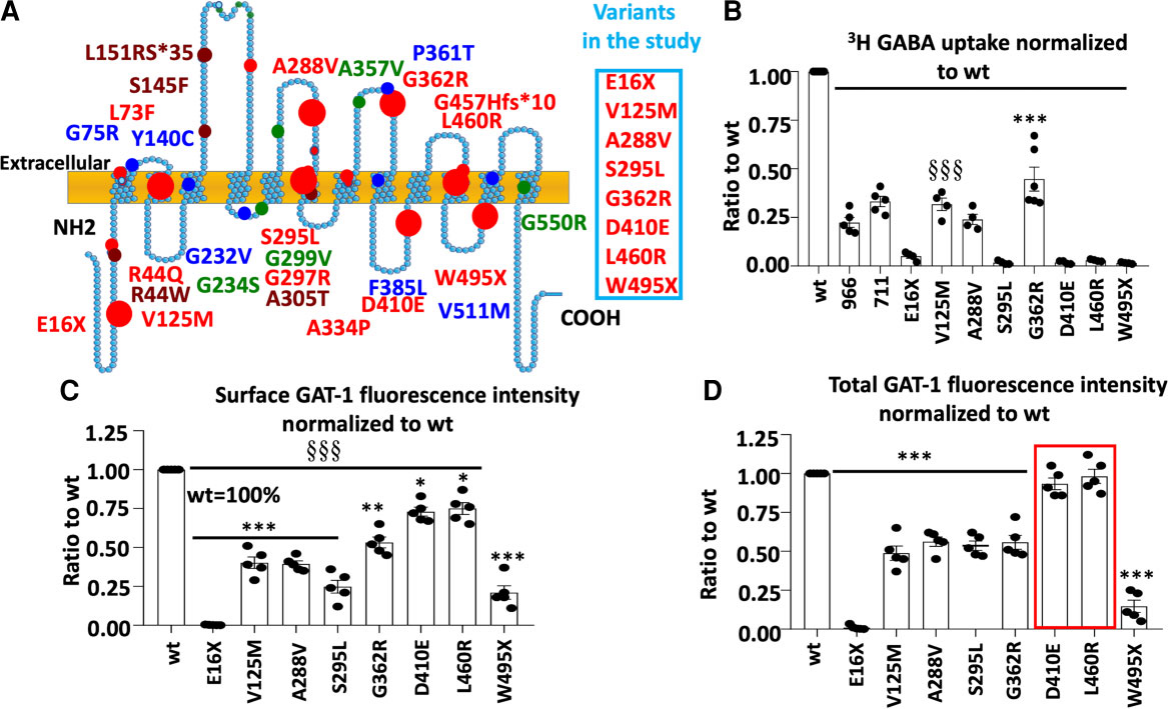
**Reduced function and trafficking of the mutant GABA transporter 1 encoded by *SLC6A1* variants associated with epilepsy, autism, ADHD and intellectual delay.** (**A**) Schematic presentation of mutant GABA transporter 1 (GAT-1) protein topology and locations of representative variants in human *SLC6A1* associated with various epilepsy syndromes and neurodevelopmental disorders as described in our previous work. These variants are distributed in various locations and domains of the encoded GAT-1 protein peptide. The large dots represent the eight variants evaluated in the study. (**B–D**) HEK293T cells were transfected with the wildtype or the mutant GAT-1^YFP^ for 48 h. (**B**) The graph represents the altered GABA reuptake function of the mutant GAT-1 encoded by eight different *SLC6A1* variants in HEK293T cells measured by the high-throughput ^3^H radio-labelling GABA uptake on a liquid scintillator with QuantaSmart. Around 966 stands for the wildtype treated with GAT-1 inhibitor Cl-966 (50 µM) and NNC-711 for the wildtype treated with NNC-711 (35 µM) for 30 min before preincubation. (**C, D**) The flow cytometry histograms depict the relative surface (**C**) or total (**D**) expression of the wildtype and the mutant GAT-1. The relative total expression level of GAT-1 in each mutant transporter was normalized to that obtained from cells with transfection of the wildtypes. *N* = 4–5 different transfections, δδδ *P* < 0.001 overall mutations versus wt, *P < 0.05, **P < 0.01, ***P < 0.001 versus wt, one-way analysis of variance and Newman–Keuls test. Values were expressed as mean ± SEM.

*SLC6A1* variants are distributed in various locations of the GAT-1 protein peptide and are associated with various epilepsy syndromes and neurodevelopmental disorders.^5,8,10,11,27,28^ We selected eight variants in the study as they are representative of missense and nonsense variants with premature stop codon mutations generated at the N- or C-terminus (Fig. 1A). HEK293T cells were transfected with the empty vector pcDNA, wildtype or the mutant GAT-1^YFP^ for 48 h before GABA uptake assay. All variants showed reduced GABA uptake activity (Fig. 1B), levels similar to those observed in cells expressing the wildtype GAT-1 treated with GAT-1 inhibitors Cl-966 (50 µM) or NNC-711 (35 µM; Fig. 1B). Consistently, the cell-surface expression of the GAT-1 was reduced in the mutant (Fig. 1C). However, the expression of GAT-1 was reduced in some mutations but not in the D410E and L460R variants.

It is interesting that the variants resulted in loss of GABA uptake function regardless of whether they were missense or nonsense variants in the N- or C-terminus (Fig. 1D). In all surveyed variants, the GABA uptake was <50% of the wildtype. All eight variants had reduced surface expression with varying magnitude as evaluated by a high-throughput assay, flow cytometry (0.002 for E16X, 0.402 for V125M, 0.394 for A288V, 0.248 for S295L, 0.532 for G362R, 0.73 for D410E, 0.75 for L460R and 0.21 for W495X versus wt = 1). Most variants had reduced total protein expression except D410E and L460R (0.01 for E16X, 0.488 for V125M, 0.562 for A288V, 0.536 for S295L, 0.558 for G362R, 0.93 for D410E, 0.98 for L460R and 0.146 for W495X). This suggests that there is a correlation in the reduction of surface protein and total protein but no direct correlation to the GABA uptake function, as some mutant protein is trafficking competent but is non-functional or has altered function.

### SLC6A1 mutations produced less mature GAT-1 protein but more ER retained immature GAT-1 protein

**Figure 2:**
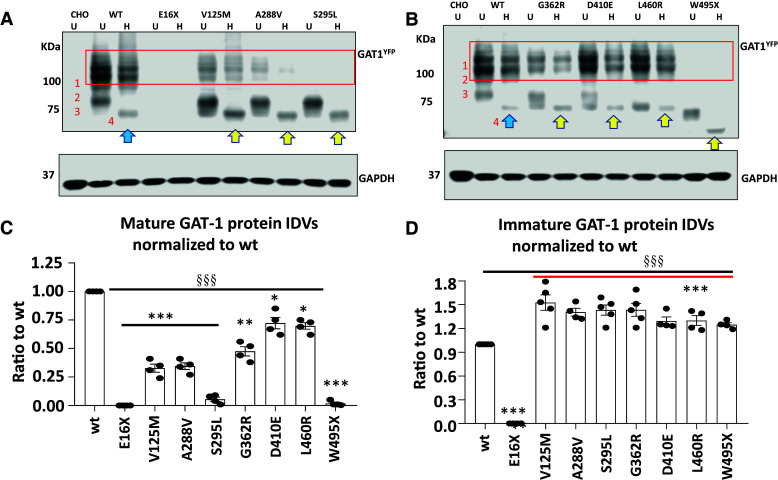
**All surveyed mutant GAT-1 transporters had less mature but more immature form of the GAT-1 protein.** (**A, B**) The total lysates of HEK293T cells expressing the wildtype or variant GAT-1 were undigested (U) or digested with Endo-H (H) and then analysed by SDS-PAGE. The membrane was immunoblotted with a rabbit anti-GAT-1. The boxed region represents the mature form of GAT-1 in cells. CHO stands for Chinese hamster ovary cells. CHO cells were used for control because of the low level of the endogenous GAT-1 expression. (**C**) The graph represents the normalized integrated protein density values (IDVs) of the mature form of GAT-1 defined by being Endo-H resistant normalized to the wildtype mature form of GAT-1 (bands 1 + 2). (**D**) The graph represents the normalized IDVs of the immature form of GAT-1 defined by being Endo-H unresistant (shifted to a lower level after H digestion) normalized to the wildtype immature form of GAT-1 (Band 3 shifted to Band 4). *N* = 4–5 different transfections, δδδ *P* < 0.001 overall mutations versus wt, *P < 0.05, **P < 0.01, ***P < 0.001 versus wt, one-way analysis of variance and Newman–Keuls test. Values were expressed as mean ± SEM.

The reduced GABA uptake activity could be caused by reduced cell surface expression or the altered gating kinetics of the transporter channels. Based on our studies on *SLC6A1* and GABA_A_ receptor epilepsy mutations, the reduction in cell surface expression of the mutant protein is the major mechanism and is mainly due to ER retention of the misfolded mutant protein, while the altered gating is a minor mechanism. Based on our studies on GABA_A_ receptors, only those proteins that are mature for glycosylation and that have trafficked beyond the ER to reach the cell surface and synapse can function.^1,11^ We thus determined the maturity of the mutant GAT-1 with Endo-H digestion, which removes the ER added glycan but not the glycan added beyond ER. As reported in our previous study,^2^ the GAT-1^YFP^ protein runs with three bands (Bands 1, 2 and 3) at 108, 96 and 90 kDa respectively (Fig. 2A and B, Supplementary Fig. 1 for full-length blots). Endo-H treatment removes the glycan added in ER but not those added beyond ER. Thus, the ER retained protein run at a lower molecular mass (Band 4). The Bands 1 and 2 are classified as mature forms of GAT-1, while the Band 3 and the down-shifted Band 4 are classified as immature GAT-1. The GAT-1(E16X) mutant protein was undetectable likely because of fast degradation of the small peptide due to the early premature stop codon. The GAT-1(W495X) mutation ran at a more reduced molecular mass due to the premature stop codon generated by the nonsense mutation (Fig. 2B). Most of the mutant GAT-1 had reduced mature form of GAT-1 (0.00 for E16X, 0.327 for V125M, 0.345 for A288V, 0.05 for S295L, 0.475 for G362R, 0.72 for D410E, 0.69 for L460R and 0.019 for W495X) but increased Endo-H-sensitive immature form of GAT-1 (0.00 for E16X, 1.526 for V125M, 1.405 for A288V, 1.432 for S295L, 1.434 for G362R, 1.293 for D410E, 1.30 for L460R and 1.248 for W495X; Fig. 2C and D), suggesting ER retention of the mutant transporter, as in our previous studies on both GABA_A_ receptor and transporters.

Levels of the mature form of GAT-1 in GAT-1(V125M, A288V, S295L and G362R) are correlated with the level of GABA uptake function. The mature form of GAT-1(D410E) and GAT-1(L460R) in HEK 293T cells does not correlate with the GABA uptake function. The GAT-1(E16X) and GAT-1(W495X) had almost no mature GAT-1 protein (Fig. 2C). By contrast, the immature GAT-1 shifted with Endo-H was higher in all surveyed mutant GAT-1 except GAT-1(E16X) as the short, truncated protein peptide in the mutant protein may be subjected to fast disposal inside ER. It is worth noting that the immature form of the mutant GAT-1 for V125M, A288V, S295L, G362R, D410E and L460R either displays a smear or run at a lower molecular mass, suggesting delayed glycosylation due to protein misfolding.

### PBA increased GABA uptake in the wildtype and the mutant transporters in HEK293T cells

**Figure 3:**
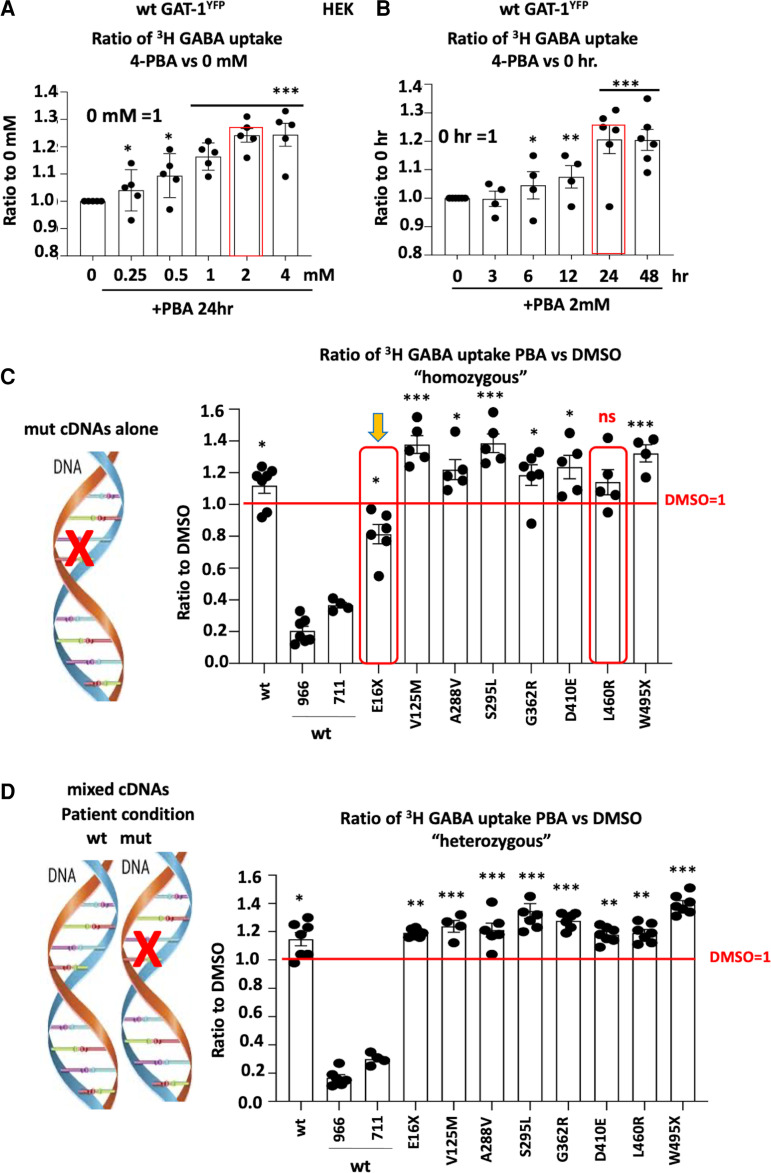
**4-Phenylbutyric acid (PBA) concentration and time dependently increased the GABA uptake in cells expressing the wildtype or the mutant GAT-1.** (**A, B**) HEK293T cells were transfected with wildtype GAT-1^YFP^ (wt) for 48 h, PBA (2 mM) was applied dropwise to each dish at different concentrations (**A**) and incubated for time durations (**B**). PBA from stocking solution (2 M) was diluted with DMEM 100 µL to desired concentration. (**A**) The graphs represent the altered GABA reuptake function of the wildtype GAT-1^YFP^ in HEK293T cells treated with PBA for a series of different concentrations. The GABA uptake activity of cells treated with PBA of different concentrations was normalized to the sister cultures treated with DMSO alone for 24 h. (**B**) The graphs represent the altered GABA reuptake function of the wildtype GAT-1 in HEK293T cells treated with PBA for a series of different time duration over treated with DMSO alone for 24 h. GABA uptake activity was measured by the high-throughput ^3^H radio-labelling GABA uptake on a liquid scintillator with QuantaSmart. (**C**) Cartoon showing the mutant allele only was expressed. HEK293T cells were transfected with the wildtype or the mutant GAT-1^YFP^ cDNAs alone for 48 h. (**D**) Cartoon showing the coexistence condition of the wildtype and the mutant allele in patients and both the wildtype and the mutant alleles were expressed. HEK293T cells were transfected with the wildtype GAT-1^YFP^ alone or in mixture of the wildtype or the mutant cDNAs for 48 h. In the mixed condition, the ratio of the wildtype GAT-1 with pcDNA or the mutant cDNAs are 1:1 with the total cDNA amount of 0.5 µg. PBA (2 mM) was applied for 24 h, while DMSO was applied as control. Both wt and the mutant were normalized to its own DMSO-treated conditions. Around 966 stands for the wildtype treated with Cl-966 (50 µM), while 711 stands for NNC-711 (35 µM). *N* = 4–7 different transfections. In **A** and **B**, *P < 0.05, **P < 0.01, ***P < 0.001 versus wt 0. In **C** and **D**, *P < 0.05, **P < 0.01, ***P < 0.001 versus its own DMSO treated. In **C**, ns stands for no significance. One-way analysis of variance and Newman–Keuls test. Values were expressed as mean ± SEM.

We have previously demonstrated that ER retention of the mutant protein can exacerbate the disease phenotype by preventing the efficient trafficking of the wildtype subunits.^8,12^ We compared PBA with other small molecules or chaperones such as menthol that could potentially modulate protein trafficking and increase GABA uptake (Supplementary Fig. 2, data not shown). We identified that PBA is effective for restoring GABA uptake among other tested compounds (data not shown). We then focused on testing the effect of PBA on rescuing the wildtype and the mutant GAT-1 activity. We first determined the effect of PBA on the wildtype GAT-1. The HEK 293T cells were transfected with GAT-1^YFP^ for 48 h and PBA was applied with a series of concentrations from 0 to 4 mM (Fig. 3B) and time durations from 0 to 48 h (Fig. 3A) before evaluation. PBA concentration and time dependently increased the GABA uptake activity (Fig. 3A and B). However, the effect of PBA at 2 mM and 24 h was the most optimal (1.24 ± 0.05 for 24 h and 1.24 ± 0.09 for 4 mM versus Wildtype 0), as occasional cell loss was observed in cells treated with PBA at 4 mM and/or 48 h. We thus chose a PBA treatment of 2 mM and 24 h for the following experiments.

Since all patients carrying *SLC6A1* variants are heterozygous and only one allele is affected in patients as illustrated (Fig. 3C), we thus tested the effect of PBA in the cells expressing the mutant cDNAs alone (‘homozygous’; Fig. 3C) or a mixture of the wildtype and the mutant cDNAs (‘heterozygous’; Fig. 3D). The cells expressing the mutant alone, PBA did not increase GABA uptake for the GAT-1(E16X) and GAT-1(L460R) mutations (0.813 ± 0.06 for E16X, 1.378 ± 0.056 for V125M, 1.22 ± 0.063 for A288V, 1.386 ± 0.058 for S295L, 1.185 ± 0.065 for G326R, 1.236 ± 0.074 for D410E, 1.14 ± 0.07 for L460R and 1.323 ± 0.054 for W495X; Fig. 3C). However, PBA increased the mutant GAT-1 activity in all tested variants except the GAT-1(E16X) in the ‘heterozygous’ condition, which reflects the patient condition in which a wildtype allele is present alongside the mutant allele (1.149 ± 0.025 for E16X, 1.33 ± 0.05 for V125M, 1.248 ± 0.053 for A288V, 1.318 ± 0.062 for S295L, 1.262 ± 0.026 for G362R, 1.17 ± 0.035 for D410E, 1.214 ± 0.037 for L460R and 1.305 ± 0.0296 for W495X; Fig. 3D). The failure to increase GABA activity in GAT-1(E16X) when expressed alone is most likely because of the very short peptide resulting from the early truncation. This renders it very misfolded while lacking ability to be refolded, thus being subjected to quick degradation inside the ER. In the ‘heterozygous’ condition, in which the cells are transfected with mixed wildtype and mutant GAT-1 cDNAs, all the mutant conditions had increased GABA uptake activity compared with the DMSO-treated conditions. This is likely due to the increased wildtype allele function. This suggests that PBA can have a broad application across an array of variants when in the heterozygous state. It is thus possible that all the patients can benefit from PBA, even in a case like GAT-1(E16X) when the mutant protein cannot be rescued.

### PBA increased GABA uptake in the patient iPSC-derived astrocytes and neurons

**Figure 4:**
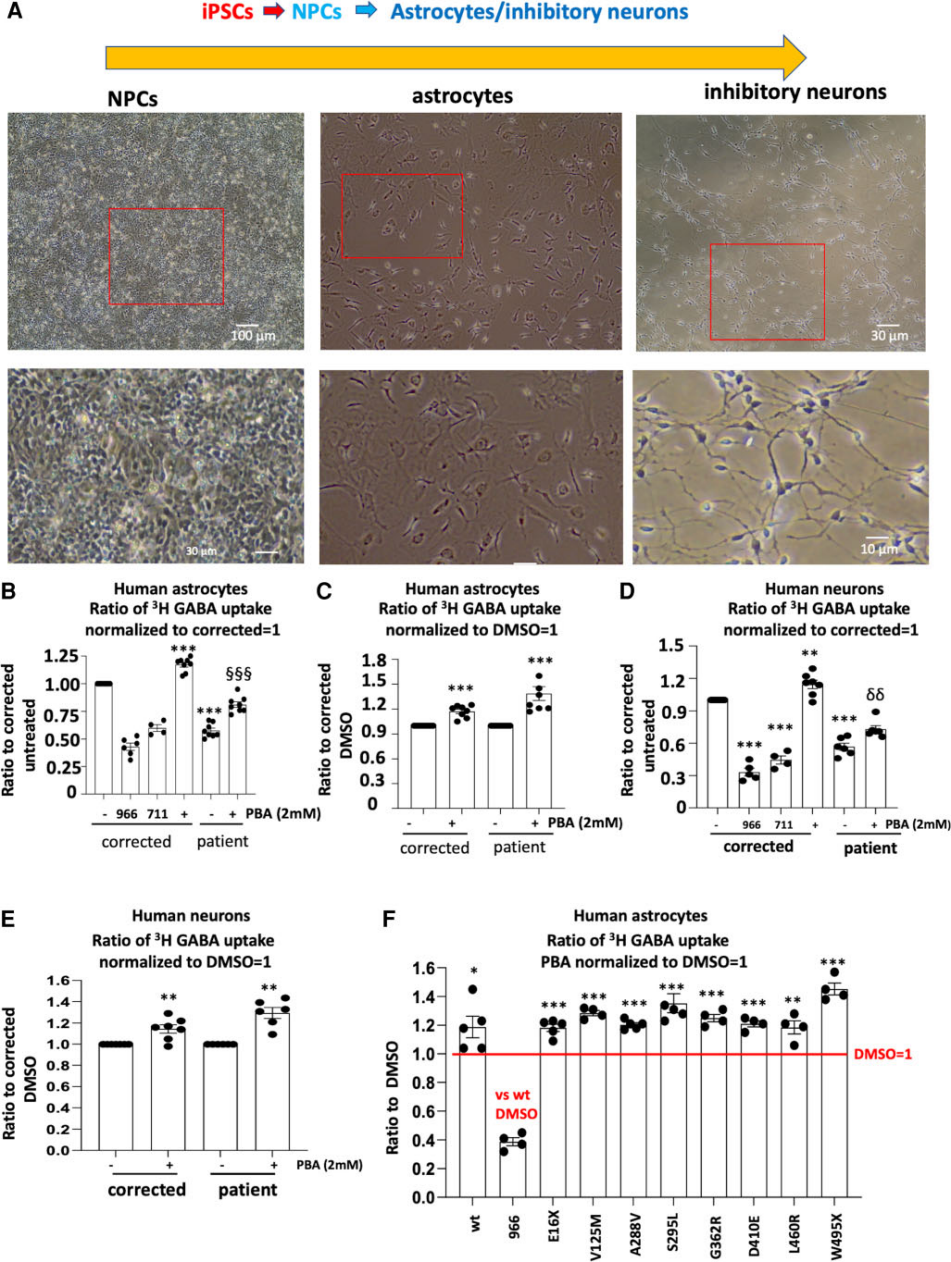
**4-Phenylbutyrate acid (PBA) rescued the GABA uptake function in the patient astrocytes and neurons.** (**A**) Human astrocytes and inhibitory neurons were differentiated from the neural progenitor cells (NPCs) from the patient and the CRISPR corrected isogenic control line. Live images of human NPCs, astrocytes and GABAergic inhibitory neurons differentiated from the human-induced pluripotent stem cells (iPSCs) on the day of experiment. (**B–E**) Astrocytes at Days 30–35 (**B, C**) or neurons at Days 60–65 (**D, E**) after differentiation were treated with PBA (2 mM) for 24 h before ^3^H radioactive GABA uptake assay. The DMSO-treated corrected or patient cells were taken as 1. (**F**) Corrected astrocytes at Days 30–35 after differentiation were transfected with the wildtype or the mutant GAT-1^YFP^ cDNAs (1 µg per a 35 mm^2^ dish) for 48 h before ^3^H radioactive GABA uptake assay. PBA (2 mM) was applied for 24 h before GABA uptake assay experiment. GABA flux was measured after 30 min transport at room temperature. The influx of GABA, expressed in pmol/µg protein/min, was averaged from duplicates for each condition and for each transfection. The average counting was DMSO-treated condition taken as 1. In **B**, **D** and **F**, 966 stands for Cl-966 (50 µM) and 711 stands for NNC-711 (35 µM) that was applied 30 min to the astrocytes transfected with the wildtype GAT-1^YFP^ before preincubation and removed during preincubation. In **B** and **D**, ***P < 0.001 versus corrected DMSO treated. §§<0.01, §§§*P* < 0.001 versus patient DMSO treated. In **C** and **E**, the PBA-treated corrected or patient GABA uptake was normalized to its DMSO treated. In **C, E** and **F**, the PBA-treated corrected/wildtype or patient/mutant GABA uptake was normalized to its DMSO treated. *P < 0.05; **P < 0.01; ***P < 0.001 versus DMSO treated in its own group. In **B**, **C**, **D** and **E**, *n* = 4–8 batches of cells. In **F**, *n* = 4–5 different transfections. Unpaired *t* test was used for **C** and **E**. One-way analysis of variance followed by a Dunnett post hoc multiple comparison test was used in **B**, **D** and **F**. Values were expressed as mean ± SEM.

We then determined if PBA could restore the GAT-1 activity in human patient iPSC-derived astrocytes and neurons. We focused on astrocytes, as there is a direct correlation of astrocytic GAT-1 deficit with thalamic absence seizures.^24,25^ We first differentiated the iPSCs to NPCs and then differentiated the NPCs into astrocytes or inhibitory neurons as in our previous study.^9^ Human astrocytes derived from iPSCs around 30 days after differentiation were treated either with DMSO or with PBA (2 mM) for 24 h before the GABA uptake assay. Based on our protocol, >90% of astrocytes adopted typical star-like astrocytic morphology after Day 27 of differentiation (Fig. 4A). Similarly, after 2 months of differentiation, ∼90–95% of cells adopted typical GABAergic interneuron morphology.^9^ When compared with the corrected isogenic control cell line, the patient astrocytes showed reduced GABA uptake activity as we previously reported.^9^ However, PBA (2 mM) treatment for 24 h increased the activity in both isogenic cells and patient cells, increasing the uptake of the patient cells from 57% to 81% of the corrected cells (Fig. 4B). The increased magnitude is larger in the mutant than the isogenic control astrocytes (1.173 ± 0.026 for control versus 1.387 ± 0.08 for patient; Fig. 4C).

We then determined the effect of PBA in human iPSC-derived neurons (Fig. 4D and E) and identified a ∼20% increase of GABA uptake in PBA-treated neurons. Since PBA caused similar upregulation on GABA uptake in both astrocytes and neurons, we thus focused on astrocytes because of their direct involvement in seizure activity,^24,25^ and tested the effect of PBA on all eight mutations in this study in human astrocytes.

We transfected the wildtype or the mutant GAT-1^YFP^ (1 µg cDNAs per 35 mm^2^ dishes) in the astrocytes derived from the corrected iPSCs for 48 h. GABA uptake activity was determined in astrocytes treated with DMSO or 2 mM PBA for 24 h. GAT-1 inhibitor Cl-966 (50 µM) or NNC-711 (35 µM) was applied to confirm that decreased radioactive uptake correlated with decreased GAT-1 functioning. PBA increased GABA activity in the wildtype and all mutant conditions (1.188 ± 0.075 for wt; 1.182 ± 0.025, E16X, 1.285 ± 0.018 for V125M, 1.204 ± 0.031 for A288V, 1.353 ± 0.065 for S295L, 1.25 ± 0.023 for G362R, 1.21 ± 0.022 for D410E, 1.18 ± 0.045 for L460R and 1.45 ± 0.04 for W495X). However, the GABA activity was increased the most in the astrocytes expressing the mutant GAT-1(W495X) (Fig. 4F).

### The human iPSC-derived astrocytes expressing mutant GAT-1(S295L) caused retention of the wildtype GAT-1 inside the ER, suggesting a dominant negative effect

**Figure 5:**
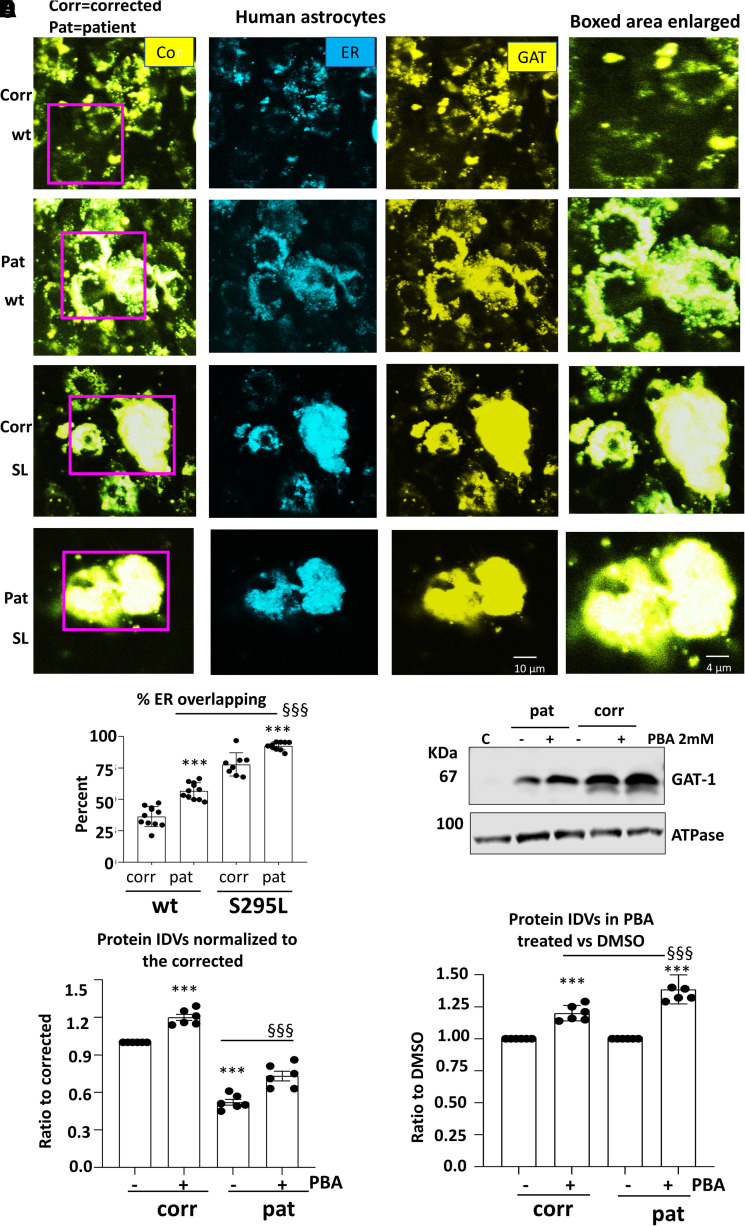
**The patient astrocytes caused ER retention of the wildtype and exacerbated the mutant GAT-1 ER retention while 4-phenylbutyrate acid (PBA) increased GAT-1 protein expression.** (**A**, **B**) Human patient corrected (isogenic control, Corr) or uncorrected patient (Pat) astrocytes at Days 30–35 after differentiation from iPSCs were co-transfected with the endoplasmic reticulum (ER) marker ER^CFP^ in combination with the wildtype or the mutant GAT-1^YFP^ cDNAs (0.5 µg:0.5 µg per a 35 mm^2^ dish) for 48 h before confocal microscopy analysis. Confocal images were acquired in live astrocytes under 63X objective with zoom under 2.5. (**A**) Boxed regions were enlarged. (**B**) The graph represents the ER overlapping signal of GAT-1^YFP^ analysed by Metamorph. (**C**, **D**) Total cell lysates from astrocytes cultured in 100 mm^2^ dishes treated with DMSO or PBA (2 mM) for 24 h and were analysed with SDS-PAGE. Membranes were blotted with a rabbit polyclonal atnti-GAT-1 antibody (**C**). The lysates of Chinese hamster ovary (CHO) cells were used as control. (**D**, **E**) The protein IDVs of the corrected or patient GAT-1 in human astrocytes were normalized to the corrected astrocytes treated with DMSO, the GAT-1 protein was normalized to its own internal control ATPase or GAPDH and then to the DMSO-treated corrected levels, which is arbitrarily taken as 1 (**D**) or its own genotype but DMSO treated, which is taken as 1 (**E**). Values were expressed as mean ± SEM. In **B**, *N* = 8–11 culture replicates. In **C**, **D** and **E**, *N* = 6 batch of cells. In **B**, ***P < 0.001 versus corrected. §§§*P* < 0.001 versus wt in patient cells. In **D**, ***P < 0.001 corrected untreated. §§§*P* < 0.001 versus patient untreated. In **E**, ***P < 0.001 versus its own untreated; §§§*P* < 0.001 versus corrected PBA treated, two-way analysis of variance followed by Bonferroni multiple comparison test.

We have previously demonstrated that GAT-1(S295L) was retained inside the ER.^9^ However, it is unknown if the mutant GAT-1 would suppress the wildtype GAT-1 via aberrant oligomerization of the wildtype GAT-1 with the mutant GAT-1. We then compared the GAT-1 expression pattern in the corrected or the patient iPSC-derived astrocytes. Human astrocytes derived from iPSCs 27–30 days after differentiation were cotransfected with the wildtype or the mutant GAT-1 cDNAs with the ER marker ER^CFP^ (Fig. 5A, Supplementary Fig. 3A). To evaluate the subcellular localization of the mutant GAT-1, we measured the colocalization fluorescence of the GAT-1-representing YFP and ER-representing CFP in the corrected or the patient astrocytes coexpressing the wildtype GAT-1^YFP^ or the mutant GAT-1^YFP^ with ER^CFP^. The mutant GAT-1(S295L) had an increased overlapping fluorescence signal with ER than the wildtype. Importantly, the wildtype GAT-1 expressed in the patient astrocytes had a greater ER overlapping fluorescence signal than the corrected cells (36.29 ± 2.4% for GAT-1^YFP^ in corrected cells, 56.6 ± 1.95% for GAT-1^YFP^ in patient cells, 77.78 ± 3.26% for GAT-1(S295L)^YFP^ in corrected cells, 92.49 ± 0.99% for GAT-1(S295L)^YFP^ in patient cells; Fig. 5B). It is worth noting that there were fewer cells with positive yellow fluorescence signal despite the large lumps of YFP in the patient cells (Supplementary Fig. 3A and B).

We then determined if PBA could increase the GAT-1 protein expression in the human astrocytes and found that PBA treatment increased the GAT-1 expression in both the corrected and the patient conditions (1.20 ± 0.024 for correct versus 1.39 ± 0.046 for patient; Fig. 5C–E, Supplementary Fig. 4 for full-length blots). The total GAT-1 protein of the patient astrocytes was increased from 52.2 to 73.2% of the corrected. We could not determine the surface GAT-1 expression because of the low yield of protein in astrocytes. It is likely that the increased GAT-1 in the patient astrocytes is due in part to the wildtype GAT-1. More detailed characterization with specific tags in GAT-1 to distinguish the wildtype versus the mutant allele may further elucidate the contribution to increased GAT-1 from each allele.

### PBA increased GABA uptake in the astrocytes and neurons in *SLC6A1^+/A288V^* and *SLC6A1^+/S295L^* knockin mice

**Figure 6:**
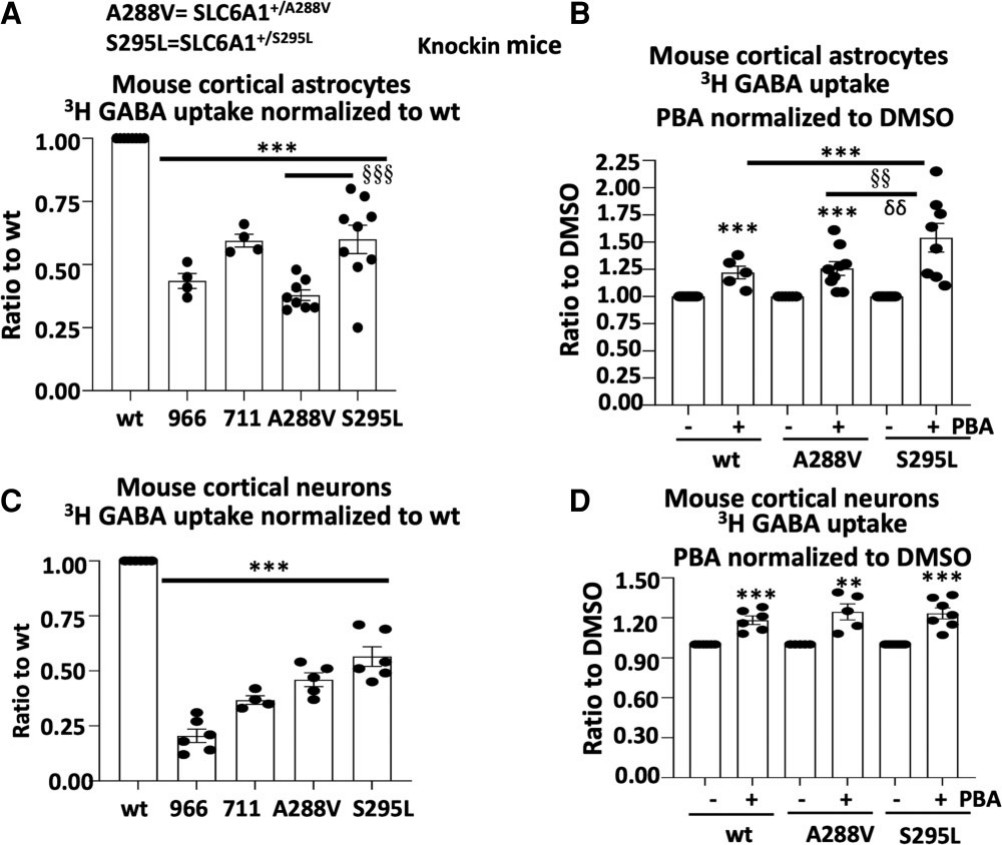
**4-Phenylbutyrate acid (PBA) rescued the GABA uptake in cortical astrocytes and neurons in *SLC6A1^+/A288V^* and *SLC6A1^+/S295L^* mice.** Mouse cortical astrocytes or neurons were cultured from postnatal pus at Days 0–3 for astrocytes and Day 0 for neurons from the *SLC6A1^+/A288V^* or *SLC6A1^+/S295L^* mouse line. (**A, B**) Astrocytes under Passage 2 were grown in 100 mm^2^ dishes and passaged into 35 mm^2^ dishes before GABA uptake assay. (**C, D**) Neurons were directly cultured in the 35 mm^2^ dishes and GABA uptake was evaluated between Days 15 and 17 after culture. Cl-966 (50 µM) and NNC711 (35 µM) were applied 30 min before preincubation and removed during preincubation. GAT-3 inhibitor SNAP5114 (30 µM) was applied during GABA uptake to make sure only GAT-1 activity was measured. The wildtype astrocytes (**A**, **B**) or cortical neurons (**C**, **D**) of either *SLC6A1^+/A288V^* or *SLC6A1^+/S295L^* were taken as 1. The cultures derived from each mutant mouse were compared with the culture from its own wildtype littermates. In **B** and **D**, sister cultures of astrocytes or neurons from different mouse lines were incubated with DMSO or PBA 2 mM for 24 h before GABA uptake. The wildtype data were pooled from two mouse lines. The GABA uptake activity of PBA treated was normalized to its own DMSO conditions. The graph represents the relative GABA uptake level normalized to cells of its own genotype treated with DMSO. Cl-966 (50 µM) or NNC-711 (35 µM) was applied 30 min before preincubation and removed during preincubation. Two-way analysis of variance followed by Bonferroni multiple comparison test was used. Values were expressed as mean ± SEM. In **A** and **C**, ***P < 0.001 versus wt; §§§*P* < 0.001 S295L versus A288V. In **B** and **D**, ***P < 0.001 versus untreated; §§*P* < 0.01 S295L versus wt treated; δδ *P* < 0.01 versus A288V treated. *N* = 4–9 batches of astrocytes from four pairs of littermates for **A**. *N* = 5–8 batches of astrocytes from four pairs of littermates for **B**; *N* = 4–7 batches of neuron cultures from 5 litters of A288V and 6 litters of S295L in **C** and **D**.

We then determined the effect of PBA in both astrocytes and neurons from both *SLC6A1**^+/A288V^*** and *SLC6A1**^+/S295L^*** knockin mice. We chose to investigate A288V and S295L in mice due to the extensive characterizations of the two variants *in vitro.*^9^ We used cortical neurons and astrocytes because the corticothalamic pathway is involved in absence seizures. Additionally, we found the data from the cortical astrocytes and thalamic astrocytes to be comparable (data not shown). We used the cortical tissue since the cortical tissue is more abundant than the thalamic tissue. GABA uptake activity can be impacted by both GAT-1 and GAT-3, so we treated the cells with a GAT-3 inhibitor, SNAP5114 (30 µM), to ensure only GAT-1 activity was measured. Compared with the wildtype, the astrocytes cultured from the heterozygous pups had reduced GABA uptake activity (0.38 ± 0.021 for A288V and 0.60 ± 0.06 for S295L; Fig. 6A). PBA treatment increased the GABA uptake activity in the astrocytes cultured from the wildtype and the mutant pups (1.22 ± 0.058 for wt; 1.26 ± 0.063 for A288V and 1.54 ± 0.13 for S295L; Fig. 6B). A similar pattern of GABA uptake reduction in the mutant mice and the upregulation in PBA-treated cells was observed in the cultured cortical neurons from the knockin mice (Fig. 6C and D).

### GAT-1 protein expression was globally reduced in *SLC6A1^+/A288V^* and *SLC6A1^+/S295L^* knockin mice and was partially restored by PBA

**Figure 7:**
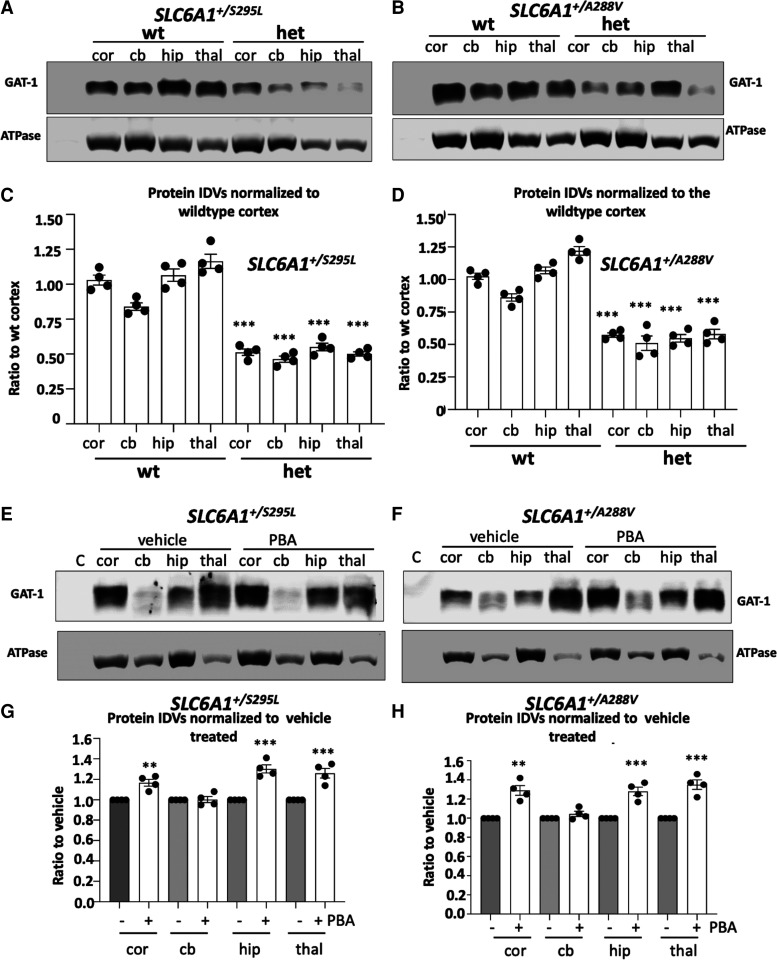
**Both *SLC6A1^+/A288V^* and *SLC6A1^+/S295L^* mice had reduced GAT-1 protein that was partially restored by 4-phenylbutyrate acid (PBA).** (**A–H**) Lysates from different brain regions [cortex (cor), cerebellum (cb), hippocampus (hip) and thalamus (thal)] from the wildtype (wt) and heterozygous (het) mice at 4–6 months old, untreated (**A**, **B**) or treated with vehicle or PBA (100 mg/kg) for 7 days (**E**, **F**) were subjected to SDS-PAGE and immunoblotted with anti-GAT-1 antibody. (**C**, **D**) Integrated density values (IDVs) for total GAT-1 from wildtype and het KI were normalized to the Na^+^/K^+^ ATPase or anti-glyceraldehyde-3-phosphate dehydrogenase (GAPDH) loading control (LC) in each specific brain region and plotted. *N* = 4 from four pairs of mice. (**G**, **H**) Integrated density values (IDVs) for total GAT-1 from het KI treated with vehicle or treated with PBA were normalized to the Na^+^/K^+^ ATPase or anti-glyceraldehyde-3-phosphate dehydrogenase (GAPDH) loading control (LC). The IDVs of the heterozygous treated with PBA were then normalized to vehicle treated. The vehicle treated in each brain region was taken as 1. *N* = 4 from four pairs of mice for **C, D, G** and **H**. Values were expressed as mean ± SEM. One-way analysis of variance or unpaired *t* test. In **C** and **D**, ***P < 0.001 versus wt, in **G** and **H**, **P < 0.01; ***P < 0.001 versus vehicle treated.

The increased GABA uptake activity could be due to increased GAT-1 protein expression caused by promoted membrane trafficking by PBA. We then determined if PBA alters the GAT-1 in knockin mice. We first profiled the GAT-1 protein expression of the mutant mice. We then treated the mice around 2 months old with either saline or PBA for 1 week. The lysates from cortex, cerebellum, hippocampus and thalamus were surveyed. Compared with the wildtype, the heterozygous mice from both the *SLC6A1^+/A288V^* and *SLC6A1^+/S295L^* mouse lines showed reduced GAT-1 expression in all surveyed brain regions (wt: 1.025 ± 0.023 for cortex, 0.86 ± 0.03 for cerebellum; 1.07 ± 0.03 for hippocampus; 1.22 ± 0.033 for thalamus; *Slc61^+/A288V^* het: 0.57 ± 0.0165 for cortex; 0.51 ± 0.056 for cerebellum; 0.547 ± 0.029 for hippocampus; 0.58 ± 0.037 for thalamus; *Slc61^+/S295L^* het: 0.513 ± 0.023 for cortex; 0.465 ± 0.021 for cerebellum; 0.55 ± 0.023 for hippocampus; 0.50 ± 0.016 for thalamus; Fig. 7A and B, Supplementary Fig. 5A and B for full-length blots), consistent with previous findings *in vitro* that the GAT-1(A288V) and GAT-1(S295L) mutations cause ER retention of the mutant protein, consequently leading to enhanced degradation.^9^ Compared with vehicle treatment, PBA treatment increased GAT-1 expression in the cortex, hippocampus and thalamus after normalization with the housekeeping protein ATPase. PBA increased the GAT-1 expression in the cortex, hippocampus and thalamus, although this was not observed in the cerebellum (*Slc61^+/A288V^* PBA: 1.29 ± 0.05 for cortex, 1.045 ± 0.028 for cerebellum; 1.28 ± 0.044 for hippocampus; 1.35 ± 0.049 for thalamus; *Slc61^+/S295L^* PBA: 1.168 ± 0.033 for cortex; 1.03 ± 0.03 for cerebellum; 1.30 ± 0.04 for hippocampus; 1.26 ± 0.04 for thalamus versus the same brain region of the vehicle-treated mice taken as 1; Fig. 7C and D, Supplementary Fig. 5C and D for full-length blots). This may suggest that the PBA-induced increase of GAT-1 can be region specific and that the increased membrane trafficking of GAT-1 contributes to increased GABA uptake. It is worth noting that the PBA treatment did not increase GABA_A_ receptor γ2 subunit (Supplementary Fig. 6 for full-length blots), suggesting that the PBA-induced protein increase could be mutation-bearing gene specific, but this merits more elucidation.

### PBA alone reduced seizure activity in *SLC6A1^+/S295L^* knockin mice

**Figure 8:**
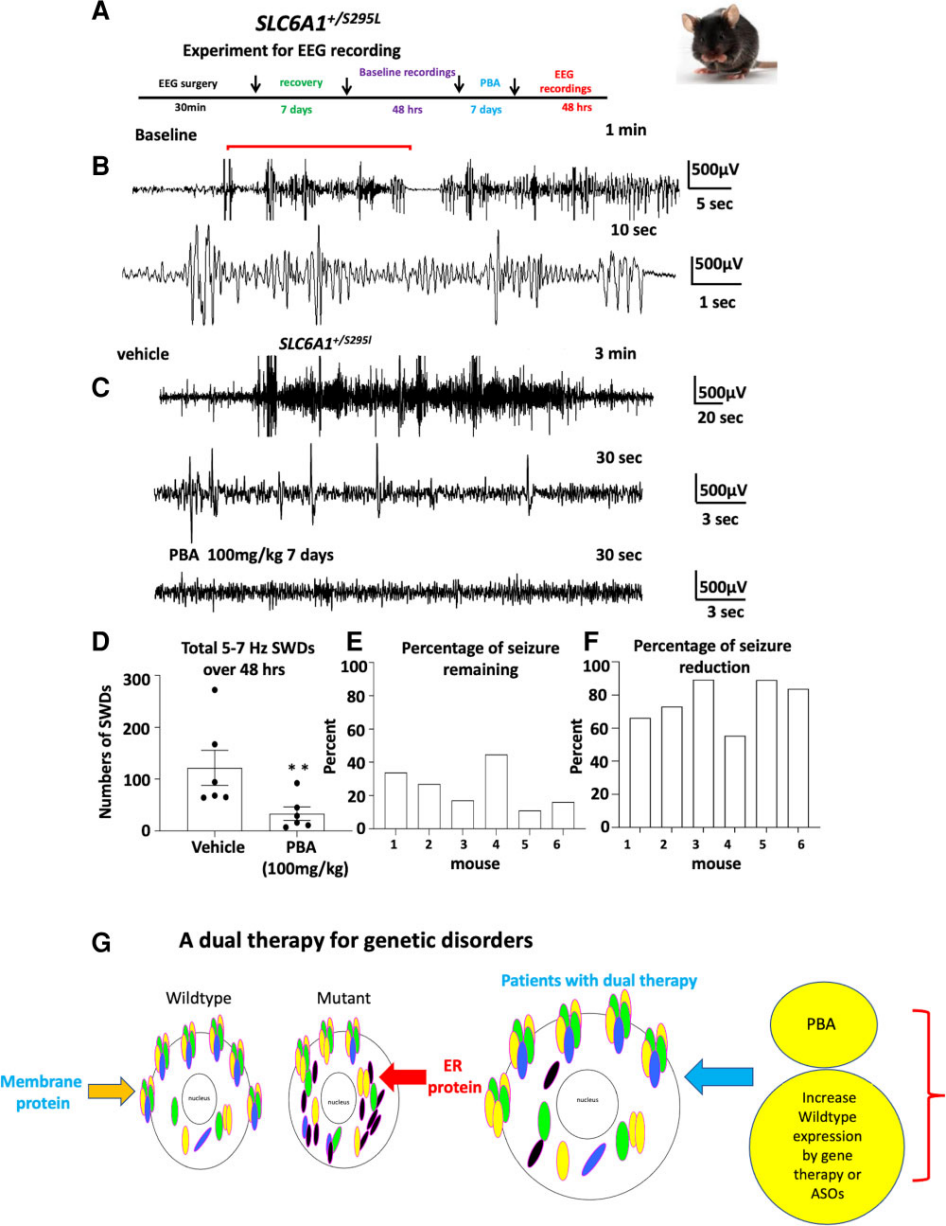
**4-Phenylbutyrate acid (PBA) alone reduced seizures in mutation knockin mice.** (**A**) Schematic depiction of experimental paradigm for EEG recordings and PBA treatment. (**B**) Representative EEG recordings show that the heterozygous *SLC6A1^+/S295L^* (het) KI mice had frequent absence like spike wave discharges (SWDs) and some myoclonic jerks during baseline recordings. (**C**) Comparison of EEG traces recorded after the vehicle (normal saline 100 µL) treated or after treatment with PBA (100 mg/kg, i.p., single dose, daily) for 7 days. (**D**) Graph showing the total number of 5–7 Hz SWDs calculated by Seizure Pro during 48 h recordings after vehicle or PBA treatment. PBA treatment (100 mg/kg, i.p., single dose, daily) for 7 days reduced seizure activity. **P < 0.01; versus vehicle treated, paired *t* test, *N* = 6 animals. Values were expressed as mean ± SEM. **(E**, **F**) Graph showing the percentage of seizure remaining (**E**) and seizure reduction (**F**) in each mouse after PBA treatment (*N* = 6 animals, paired *t* test). (**G**) We propose a dual therapy as a feasible approach for treating *SLC6A1* variants and other genetic disorders by boosting the wildtype allele, removing the mutant allele and ER stress with PBA.

A major phenotype in *SLC6A1* variant mediated disorders is epilepsy, leading us to investigate whether PBA reduces seizure activity in mice. We tested the effect of PBA in *SLC6A1^+/S295L^* mice. First, ∼2-month-old mice were implanted with EEG head mounts. After 5–7 days recovery, the mice were treated with a normal saline vehicle for 7 days, and then subject to video-monitored EEG recording for 48 h. After at least 1 day rest, the mice were administered with PBA (100 mg/kg) for 7 days followed by 48 h EEG recording (Fig. 8A). We observed frequent absence-like seizures and occasionally generalized tonic clonic seizures during routine handling in *SLC6A1^+/S295L^* mice. In EEG recordings, the *SLC6A1^+/S295L^* mice displayed frequent absence like activity (5–7 Hz) and some myoclonic jerks with or without behavioural correlation (Fig. 8B and C, Supplementary Fig. 7 for expanded traces). However, compared with vehicle-treated baseline recordings, PBA treatment reduced the 5–7 Hz SWDs from 127.7 ± 34.08 with vehicle treated to 33.3 ± 13.02 over 48 h EEG recordings (Fig. 8C and D). Importantly, every mouse had reduced seizures compared with vehicle treatment ranging from 55 to 89% seizure reduction and with 11 to 44.6% seizure remaining (Fig. 8E and F). This suggests PBA alone can reduce seizure activity in *SLC6A1^+/S295L^* mice. This is likely due to promoted membrane protein forward trafficking and increased functional GAT-1 expression, which includes both the wildtype allele and the rescuable mutant allele. It is worth noting that there was no increase in the expression of GABA_A_ receptor subunits, such as the γ2 subunit (Supplementary Fig. 6). However, the level of specificity in the upregulation of the functional GAT-1 versus other membrane proteins merits more detailed investigation.

## Discussion

### Reduced membrane trafficking due to ER retention is a major cause for *SLC6A1* variant-mediated disorders

*SLC6A1* variants are associated with a wide spectrum of neurodevelopmental disorders.^8,11,28^ We have previously studied the impact of the variants on GABA uptake function, membrane trafficking and subcellular localizations of the mutant protein in both cultured neurons and astrocytes from mouse and human iPSC-derived GABAergic neurons and astrocytes.^9^ We found that the mutant GAT-1 stemming from missense mutations resulted in complete or partial loss of GABA uptake function, while the premature codon generating nonsense mutations resulted in nearly complete loss of function.^9–11^ The mutant protein is often retained inside the ER and subject to enhanced degradation, as observed in many GABA_A_ receptor subunit variants, suggesting that *SLC6A1* variants and GABA_A_ receptor subunit variant-mediated disorders could be rescued with a common treatment targeting ER retention.

### The impaired membrane trafficking resulted in reduced functional and mature GAT-1 but increased non-functional, immature GAT-1

What are the molecular mechanisms underlying the reduced surface expression of the mutant GAT-1? For a membrane protein such as GAT-1 or GABA_A_ receptor subunits, only those proteins that are correctly folded and fully glycosylated can traffic to the cell surface or synapse, where it exerts biological function. The protein that is left inside the ER has no biological function. However, the ER-retained mutant protein can prevent the wildtype protein from correct oligomerization and trafficking, causing ER stress in the cell. Endo-H digestion can distinguish the mature form of GAT-1 versus immature GAT-1 because the ER-attached glycan is sensitive to Endo-H digestion. Based on our findings, the mutant GAT-1 resulted in decreased functional mature GAT-1 levels but increased amounts of immature ER-retained GAT-1 except for GAT-1(E16X), which is likely subject to rapid disposal. Similar ER retention of GAT-1 was reported in astrocytes in our previous studies.^9^ Therapeutic strategies that increase mature GAT-1 levels and reduce the immature GAT-1 levels should be beneficial for many mutations with this underlying mechanism.

### The mutant GAT-1 can prevent forward trafficking of wildtype GAT-1

The oligomerization status and detailed structure of membrane-bound GAT-1 remain unknown. It is possible that wildtype GAT-1 and mutant GAT-1 can form dimers or other high molecular mass protein complexes and that the trafficking-deficient mutant GAT-1 could potentially interfere with the trafficking of the wildtype. Thus, any drug that can promote protein trafficking could be beneficial to *SLC6A1* variant-mediated disorders and can be considered for further treatment development. This notion is evidenced in the human astrocytes, where wildtype GAT-1 was more ER bound when expressed in the patient cells than expressed in the corrected cells (Fig. 5). The GAT-1(S295L) protein is retained inside the ER with minimal surface expression. In the patient cells expressing the mutant GAT-1(S295L)^YFP^, the GAT-1-representing yellow fluorescence formed large clumps inside cells with enlarged ER, suggesting substantial ER retention of the mutant protein inside the ER. PBA increased GABA uptake activity and the total functional GAT-1 protein in both the corrected and patient astrocytes.

### PBA could rescue the GABA uptake activity across cell types

We tested the effect of PBA in heterologous cells, neurons and astrocytes from both mice and humans finding that PBA increased GABA uptake across cell types. It is worth noting that we observed an increase of GABA uptake in NPCs after PBA treatment, although the baseline activity of GABA uptake in NPCs is relatively low (data not shown). This is consistent with our previous study on comparison of GABA uptake in NPCs, astrocytes and inhibitory neurons.^9^ This suggests that the protein quality control machinery is conserved across species and cell types. This is important considering the very early onset of the *SLC6A1* variant-mediated disorders in human patients and the expression of GAT-1 function in multiple cell types. This indicates PBA treatment can increase GAT-1 functioning before and after the postmitotic mature neurons are formed. It also demonstrates that PBA can improve the function of GAT-1 in both progenitor cells and the derived neurons and astrocytes. Our data indicate that PBA treatment is likely disease modifying, as it can improve GAT-1 function in all involved cell types. It corrects the patho-mechanisms at a root level instead of simply masking disease symptoms, thus enable it to mitigate both seizures and comorbidities while improving disease outcome.

### PBA increased the GAT-1 expression and function in the heterozygous condition likely through the wildtype allele and in some cases, the functional mutant allele

PBA is a hydrophobic chaperone, and it may prevent the aberrant interaction of the mutant GAT-1 protein with its wildtype binding partners. For the mutant allele alone, PBA increased most mutant GAT-1 levels except those in the E16X mutation. This is likely because the GAT-1(E16X) is severely misfolded, with only 15 amino acids left in the protein peptide, and thus could not be rescued. In the heterozygous conditions with the wildtype allele present, PBA increased GABA uptake for all surveyed mutations. The increase of function in the heterozygous GAT-1(E16X) condition is likely due to the wildtype allele. In some cases, such as GAT-1(A288V), the mutant allele could also be functional if rescued and present on the cell surface. This is critical, since all *SLC6A1* variant-bearing patients identified so far are heterozygous, suggesting that they can potentially benefit from a treatment option like PBA.

Based on our data, it is likely that the effect of PBA is bidirectional (Fig. 3C). For the wildtype allele, it can promote membrane trafficking while facilitating the disposal of those severely impaired proteins such as GAT-1(E16X), thus making the trafficking more efficient for the wildtype allele. Because the patients are heterozygous, this will consequently increase the net GABA uptake at least from the wildtype even if the mutant GAT-1 cannot be rescued. This hypothesis of increasing the wildtype GAT-1 in the presence of a non-functional mutant allele was supported by our findings in human iPSC-derived cells and the heterozygous knockin mice. This suggests that PBA could potentially be beneficial for all patients carrying *SLC6A1* mutations because of the presence of the wildtype allele.

### PBA treatment reduced seizures in variant-bearing knockin mice

Seizures and abnormal EEGs are common among patients with *SLC6A1* mutations, and thus can serve as a good biomarker for evaluating the effect of PBA. GAT-1(S295L) protein alone has no GABA uptake function as described here and in our previous work.^9^ In *SLC6A1^+/S295L^* mice, we found significant loss of GAT-1 in all major brain regions. PBA treatment for 1 week reduced seizures in the *SLC6A1^+/S295L^* heterozygous mice, likely via increasing the functional GAT-1. The magnitude of GAT-1 increase is variable in different brain regions. The magnitude of increase of GABA uptake and GAT-1 protein expression is modest. Based on our studies on other epilepsy mouse models *Gabrg2^+/-^* and *Gabrg2^+/Q390X^*, a small increase of γ2 subunit reduces seizure severity from Dravet syndrome to infrequent absence or seizure free.^34,35^ This is likely true for the PBA-treated mice. The seizure burden in *SLC6A1^+/S295L^* mice was reduced by ∼76% with PBA treatment alone. This is likely via the increase of functional GAT-1 and possibly other undefined mechanisms.

Deficits in GABA uptake are a common mechanism underlying the heterogeneous clinical phenotypes associated with *SLC6A1* variants, and PBA increases GABA uptake activity. This suggests that PBA could be applied to patients with the same molecular defects regardless of clinical phenotype, including various epilepsy syndromes, autism, neurodevelopmental delay and others. PBA targets at a root level of disease pathophysiology, thus it is likely disease modifying.

### PBA is a feasible dual therapy for *SLC6A1* mutations and possibly for many other genetic disorders

There is no effective treatment for *SLC6A1* mutation-mediated disorders to date. Valproic acid has been reported to control seizures but could not improve cognition.^10^ This suggests that the impaired cognition is likely caused by the deficit of GAT-1 function instead of being a consequence of seizure activity. Although the detailed mechanisms of action for PBA needs more thorough investigation, we believe the enhanced membrane trafficking and increased functional GAT-1 levels by upregulating the wildtype allele are major mechanisms underlying the GAT-1 function restoration and seizure mitigation.

There are numerous ion channels and transporters associated with epilepsy, autism and neurodevelopmental delay. Based on our substantial characterizations of the impact of mutations in GABA_A_ receptors, and more recently in GAT-1, we propose that impaired trafficking and ER-associated degradation are common mechanisms for mutations in GABA receptors, transporters and beyond. This study, in combination with our previous work on the patho-mechanisms of GABA receptors and transporter 1 mutations, provides evidence that PBA may be a feasible treatment option and is disease modifying (Fig. 8G). Considering PBA is already FDA approved for paediatric use and is orally bioavailable, with gene therapy and antisense oligonucleotides on the horizon, this treatment could open a new door or even bring a cure when used in combination with other treatment options, for many genetic disorders. This could be achieved by boosting the wildtype functional allele via gene therapy and reducing the ER-retained mutant protein and ER stress via PBA. We propose this dual therapy as an actionable mode of treatment for *SLC6A1* variant-mediated disorders and others with similar pathophysiology.

### Promise and remaining challenges

Our data show promise in preclinical cell and mouse models, and a pilot clinical trial has been prompted by our initial findings (https://clinicaltrials.gov/ct2/show/nct04937062). However, there are many remaining concerns around using PBA. For example, what are the main mechanisms of action for PBA in restoring GABA uptake? How specific is the effect of PBA on membrane trafficking of the targeted protein? Which allele, the wildtype, or the mutant, contributes to the increased GABA uptake? What is the effect of PBA on the GAT-1 mutants, such as GAT-1(A288V), with remaining function in the presence of the wildtype allele? We propose that PBA increases the wildtype protein forward trafficking. Multiple mechanisms of action could be involved, and future studies with combined multidisciplinary approaches from *in vitro* and *in vivo* with differentially tagged wildtype and mutant alleles will elucidate the detailed mechanisms and provide answers. Nevertheless, this study first identified that PBA could be a treatment option for genetic epilepsy mediated by *SLC6A1* variants.

## Supplementary Material

fcac144_Supplementary_Data

## Abbreviations

ER =: endoplasmic reticulum
EYFP =: enhanced yellow fluorescent protein
GABA =: γ-aminobutyric acid
GABA_A_ =: γ-aminobutyric acid type A
GAT-1 =: γ-aminobutyric acid transporter 1
HEK293T =: human embryonic kidney
iPSC =: induced pluripotent stem cell
PBA =: 4-phenylbutyrate acid
SLC6 =: solute carrier
*SLC6A1* =: solute carrier Family 6 Member 1
SWDs =: spike wave discharges
