# Supplementary material for: 4-Phenylbutyrate restored γ-aminobutyric acid uptake and reduced seizures in SLC6A1 patient variant-bearing cell and mouse models
Source: Brain Commun. 2022 Jun 6;4(3):fcac144. doi: 10.1093/braincomms/fcac144 (PMC9336585; doi:10.1093/braincomms/fcac144)
Supplement: fcac144_Supplementary_Data [file fcac144_Supplementary_Data.zip › Supplementary Figures.pdf]

Supplementary Figure 1

Full-length gels for Figure 2A and 2B

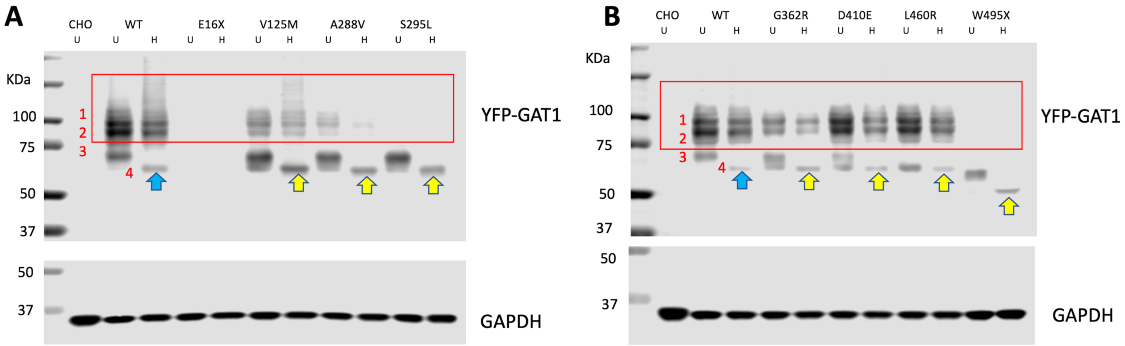

Supplementary Figure 1: full-length gels of immunoblots Figure 2A and Figure 2B.

Supplementary Figure 2

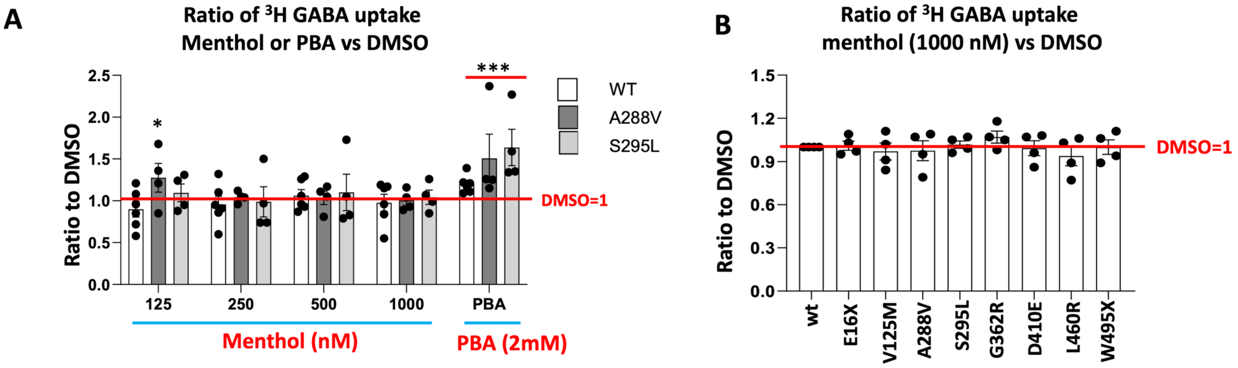

Supplementary Figure 2. Effect of other chaperones like menthol on GABA uptake. A, B. HEK293T cells were transfected with wildtype (wt) or the mutant GAT-1YFP for 48 hr., Menthol (125, 250, 500, 1000 nM) or PBA (2mM) was applied dropwise to each dish (A) and incubated for 24 hrs. (A). PBA from stocking solution (2 M) was diluted with DMEM 100 µl to desired concentration. A. The graphs represent the altered GABA reuptake function of the wildtype GAT-1 in HEK293T cells treated with PBA for a series of different concentrations. The GABA uptake activity of cells treated with PBA of different concentrations was normalized to the sister cultures treated with DMSO alone for 24 hrs. (A). The graph shows the GABA uptake function of the wildtype GAT-1 or the mutant GAT-1(A288V) and GAT-1(S295L) treated with menthol or PBA. B. The graph shows the GABA uptake function of the wildtype GAT-1 or the mutant GAT-1 treated with menthol (1000 nM). (*$p < 0.05$; ***$p < 0.001$ vs DMSO, n=4-5 transfections).

Supplementary Figure 3 Enlarged images of Figure 5A

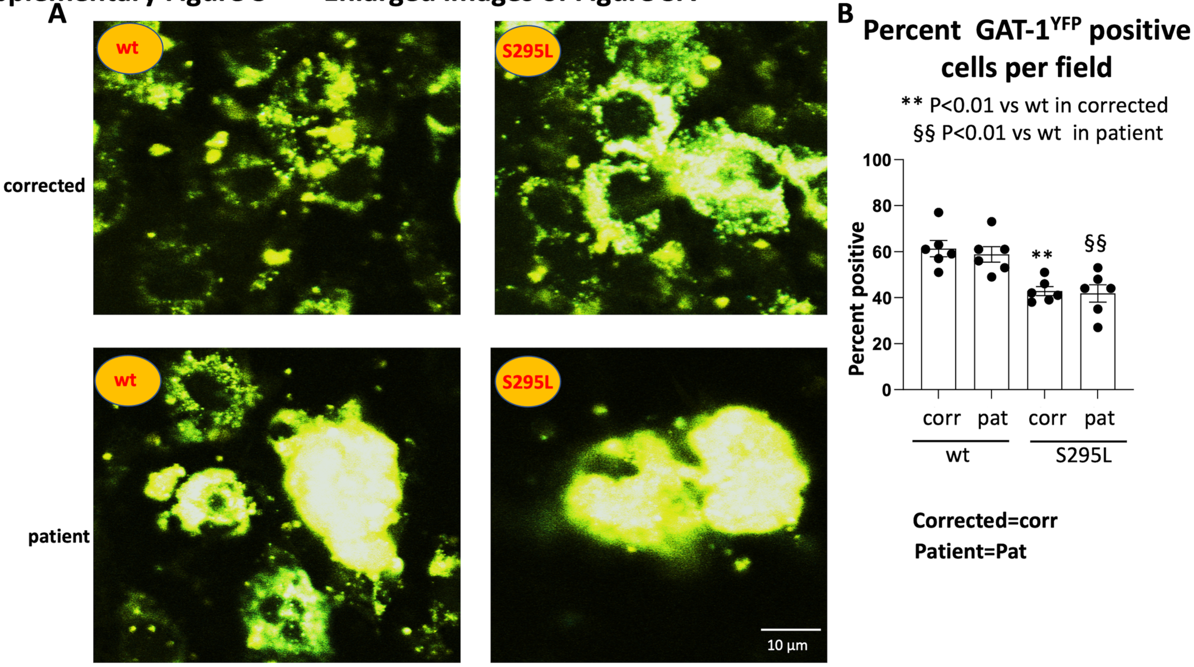

Supplementary Figure 3. Enlarged images of the wildtype and mutant GAT-1YFP in live human astrocytes (A). B. Graph showing the reduced percentage of cells expressing detectable GAT-1YFP.

Supplementary Figure 4

Full-length gels for Figure 5C

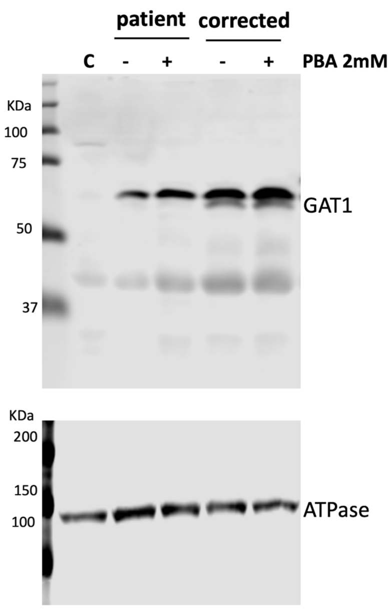

Supplementary Figure 4: Original full-length gels for Figure 5C.

Supplementary Figure 5

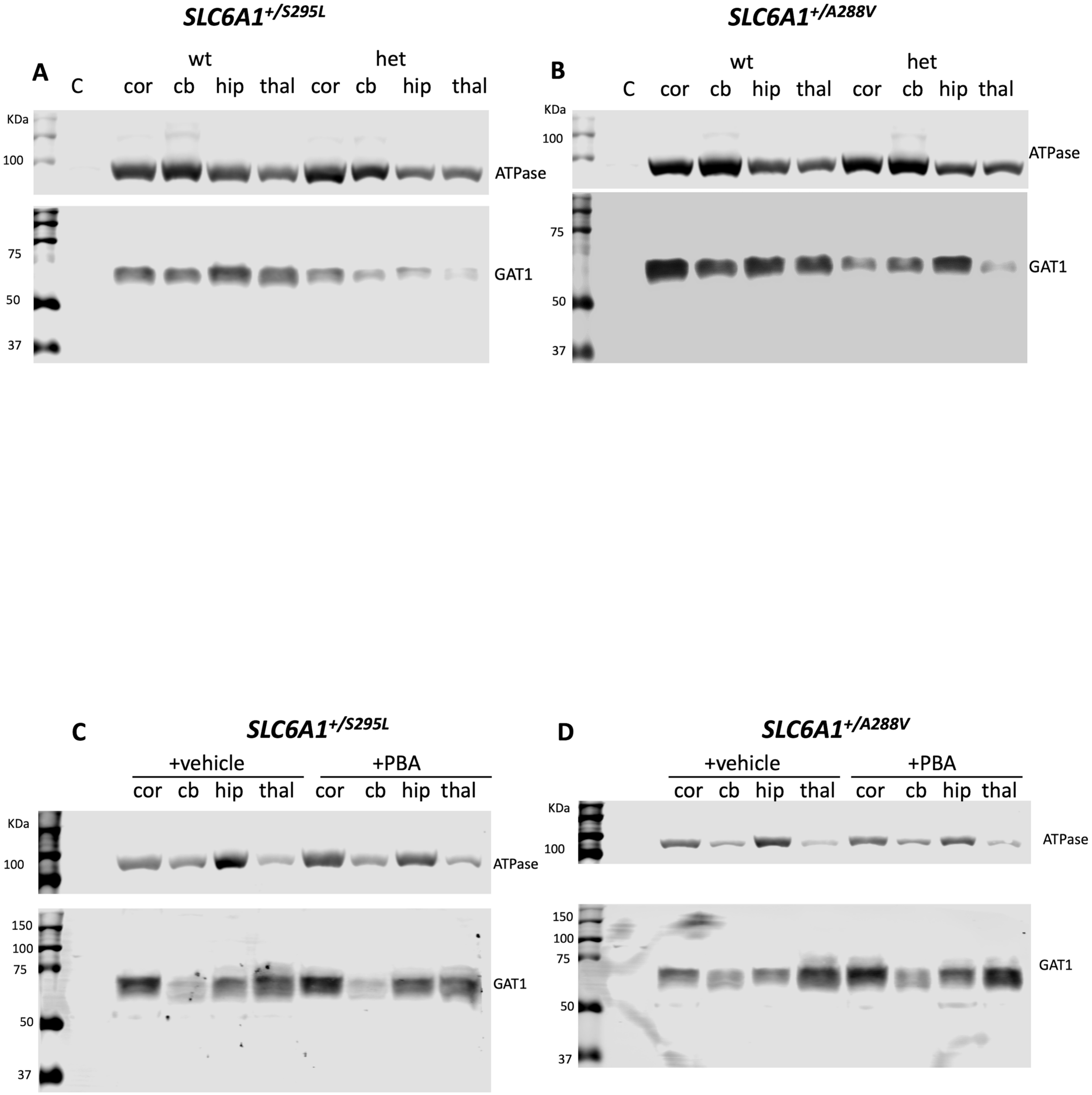

Supplementary Figure 5: Original full-length gels for Figure 7A, 7B, 7E and 7F.

Supplementary Figure 6

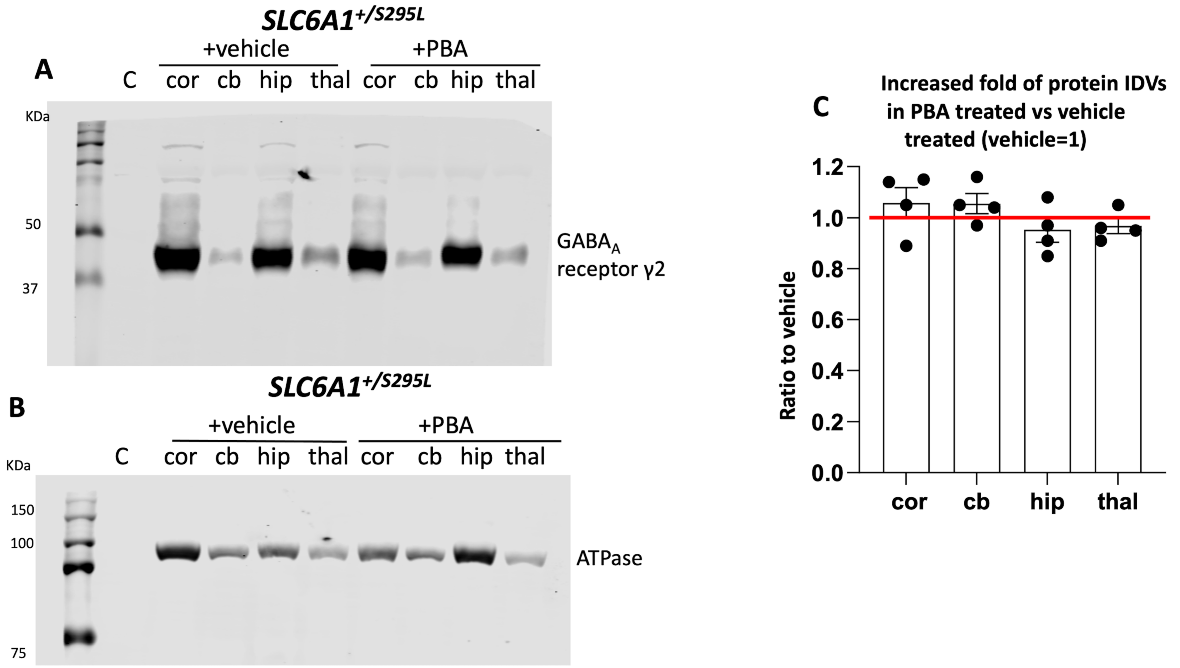

Supplementary Figure 6: The GABAA receptor γ2 subunit protein was not increased in 4-phenylbutyrate treated Slc6a1+/S295L mice. A, B. Original full-length gels for the GABAA receptor γ2 subunit protein in different brain regions of the heterozygous mice treated with vehicle or PBA. C. Graph showing the fold change of protein IDVs from mice treated with PBA normalized to its loading control ATPase and then to the vehicle treated. The heterozygous (het) mice at 2-8 months old were treated with vehicle or PBA (100 mg/kg) for 7 days. The total lysates from cortex (cor), cerebellum (cb), hippocampus (hip) and thalamus (thal) were subjected to SDS-PAGE and immunoblotted with rabbit anti-γ2 antibody. Integrated density values (IDVs) of the γ2 subunit from het mice treated with PBA were normalized to the Na+/K+ ATPase or anti-glyceraldehyde-3-phosphate dehydrogenase (GAPDH) loading control (LC) and then to the vehicle treated, which is arbitrarily taken as 1 in each specific brain region. N= 4 blots from 4 pairs of mice.

Supplementary Figure 7

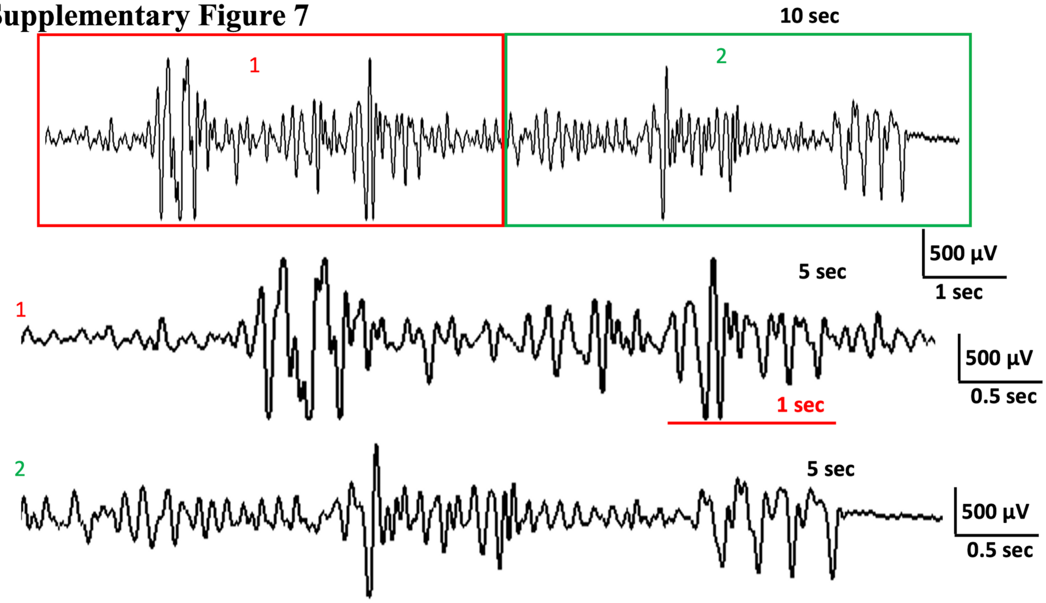

Supplementary Figure 7. Expanded EEG traces from a Slc6a1+/S295L mouse showing increased 5-7 Hz spike-wave discharges.
