# Supplementary material for: 4-Phenylbutyrate restored γ-aminobutyric acid uptake and reduced seizures in SLC6A1 patient variant-bearing cell and mouse models
Source: Brain Commun. 2022 Jun 6;4(3):fcac144. doi: 10.1093/braincomms/fcac144 (PMC9336585; doi:10.1093/braincomms/fcac144)
Supplement: fcac144_Supplementary_Data [file fcac144_Supplementary_Data.zip › Supplementary Methods.docx]

**Supplementary Methods**

***Human patient derived induced pluripotent stem cells (iPSCs), neural progenitor cells (NPCs), astrocytes and neurons.***

**Overview:** the corrected and patient cell lines were maintained in mTeSR and the plates were coated with Geltrex (1 (Geltrex stock solution):50 DMEM/F12)) overnight. Geltrex stock was prepared by adding 5ml DMEM to the original tube (5ml DMEM/F12+5ml Geltrex) and aliquoted to 300 µl per tube and stored at -20°C. The media was refreshed daily for iPSCs. The differentiation of neural progenitor cells (NPCs) was induced by STEMdiff SMADi Neural induction kit from STEMCELL.

The cortical inhibitory neurons were prepared following the protocol in previous reports ^1,2^. The neurons were used for experiment at day 60-65 after differentiation. Neurons were validated by immunostaining with NeuN, DLX, synapsin, and synaptophysin^2^. Neuronal differentiations were initiated from the NPCs at day 10 in dish of passage 2 of neural progenitor cells (NPCs) after neuronal induction. The 1^st^ passage from iPSCs for neural induction was taken as P0. The differentiation of astrocytes was started from NPC day 5 at P1. The experiments were carried out after 27 days for astrocytes or 2 months for neurons after differentiation. For experiment of each condition, at least double or triple the dishes were used each time. The differentiation of astrocytes was initiated by using the Astrocyte medium (ScienCell) for about 30 days and were passaged at ~70% confluence. The cells used for experiment were about ~80 to ~90% confluence.

**iPSCs:** iPSCs were cultured in 35mm dishes with mTeSR from Gibco. The dishes were coated Geltrex (1:50 DMEM/F12) overnight. iPSCs were passaged at ~80% confluency with 0.5mM EDTA in 1XPBS. iPSCs at passages 14-22 were used in the study.

**NPCs:** NPCs were differentiated from iPSCs at passage 22-24 with differentiation of neural progenitor cells (NPCs) was induced by STEMdiff SMADi Neural induction kit from STEMCELL. iPSCs were seeded in a Geltrex coated dish at a density of ~2 X10^6^ cells/ per one 35mm dish or 6 well plate. The medium was refreshed daily, and the cells were passaged at day 5-7.

NPCs at Passage 1 which was at least 10-12 days after neural induction were used for this study.

**Astrocytes:** Astrocytes were differentiated starting from day 5 of NPC passage 1 with the astrocyte culture medium from Sciencell (catalog 1801) following the protocol as previously described ^3^. The cell culture medium was refreshed every other day and the cells were passaged at ~70% confluency. About 1.5X 10^5^ cells were seeded in each 35mm dish or each well in 6-well plate when passaging. The differentiation was continuous until the day 30 and all the astrocytes used in experiments in this study were at day 30 after differentiation.

**GABAergic inhibitory neurons:** Cortical inhibitory GABAergic neuronal differentiation was based on previously described^1^. The differentiation started from iPSCs at passage 24-28 and was comprised of three 3 major steps including neural induction, cortical specification and GABAergic neuronal differentiation.

**Neural induction:** The same protocol used for generating NPCs for astrocytes was used for neuronal differentiation.

**Cortical specification:** At day 10-12 of neural induction, the cells were harvested with accutase (Sigma), collected by centrifugation, washed in DPBS, and subsequently seeded at a density of 3X 10 ^5^ cells in a 35 mm dish or 6-well plate coated with poly-L-ornithine (PLO)/laminin (LMN; 20µg/ml). In the condition involving cover slips, 3 cover slips were placed in each 35 mm dish. From day 10-12, NPCs were patterned towards cortical fate using NMM medium (242 ml neurobasal, 242 ml DMEM/F12+Glutamax, 2.5ml Non-essential amino acids, 3.75ml 100XGlutamax, 5.0ml penicillin-streptomycin, 2.5 ml 100XN2 supplement, 10 ml 50XB27, 312.5 µl insulin supplemented with 5 nM recombinant mouse sonic hedgehog (SHH C25II; RnD Systems),1μM SHH agonist Purmorphamine (Miltenyi biotech), 10 ng/ml re-combinant human brain derived neurotrophic factor (BDNF; Thermo-Fisher Scientific), 200μM ascorbic acid (Sigma) and 100μM2′-O-Di-butyryladenosine 3,5′-cyclic monophosphate (cAMP; Sigma). Medium was refreshed on every 3rd day.

**Maintenance and further differentiation:** After 10 days, cells started to obtain NPC morphology and were gently harvested for further maturation. Briefly, cells were plated onto PLO/LMN coated coverslips/dishes in **NMM** medium as detailed below supplemented with BDNF, ascorbic acid and cAMP. Medium was changed twice a week from this point onwards until day 60-65 starting from differentiation which is the age of the cells we use for this study.

**NMM media:** 242 ml Neurobasal, 242 ml DMEM/F12 + Glutamax, 2.5ml Non-Essential Amino Acids, 3.75 ml 100X Glutamax, 5.0 ml Penicillin-streptomycin, 2.5ml 100X N2 Supplement, 5.0 ml 50XB27 Supplement, 312.5µl Insulin (4mg/ml stock to final concentration of 2.5µg/ml). 1.75µlBME (14.3 M stock to final concentration of 50 µM).

Coating for neurons, we used PLO (Poly-L-ornithine, Sigma, 0.5mg/ml in Borate buffer) for overnight and then Laminin (20ug/ml) coating for at least 1hr.

Cell density at seeding: for iPSCs: 2-2.5 Million cells/ per 35mm dish or 6 well, for NPCs: 2-2.5 Million cells/ per 35mm dish or 6 wells; for astrocytes: 1.5X 10^5^ cells/ per 35mm dish or 6 well, For neurons: ~ 1 million cells/per 35 mm dish or 6 wells.

**Culture of human embryonic kidney (HEK293T) cells and Chinese hamster ovary (CHO) cells**

***HEK 293T cells*:** Human embryonic kidney cells were cultured as monolayers in 100 mm^2^ dishes. Cells were cultured in Dulbecco’s Modified Eagle’s Medium supplemented with 10% FBS and 1% penicillin/streptomycin in 37°C in 5% CO_2_-95% air. Cells were maintained in culture medium until appropriate confluency for experimentation.

***CHO cells:*** Chinese hamster ovarian cells were cultured as monolayers in 100 mm^2^ dishes. Cells were cultured in Dulbecco’s Modified Eagle’s-F12 Medium supplemented with 10% FBS and 1% penicillin/streptomycin in 37°C in 5% CO_2_-95% air. Cells were maintained in culture medium until appropriate confluency for experimentation.

**Culture for mouse neurons and astrocytes and transfection**

***Mouse cortical neurons****:* Mouse neurons were cultured from postnatal day 0 mouse pups. The detailed procedures for neuronal culture have been previously described^4,5^. The neurons were plated at a density of ~0.5 ×10^5^ per well in the six-well plate or 35 mm^2^ dish in plating media that contained 420 mL DMEM, 40 mL F12, 40 mL fetal bovine serum, 1 mL penicillin and streptomycin, and 0.2 mL L-Glutamine (200 mM) for 4 hrs. Neurons were then maintained in Neurobasal media that contained B27 supplement (50:1), L-Glutamine (200 mM), and 1 mL penicillin and streptomycin. The experiments for GABA uptake were directly conducted in the cultured six-well plate or the 35mm^2^ petri dishes.

***Mouse cortical astrocytes:*** Briefly, the dishes are coated with poly-lysine (0.1mg/ml) for 2 hrs at room temperature. The cortices of postnatal day 0-3 pups sometimes from the same litter used for neuronal cultures were dissected. The tissues were minced after removal of meninges and then digested with 0.25% trypsin for 10 min at 37°C. The tissues were then mechanically dispersed with a 10 ml sterile pipette and a 1000 µl tip. The mixed cell suspension was then precipitated for 3 min in a 15ml conic tube to remove the large chunks and cell debris. The mixed cell suspension was carefully aspirated and removed to another 15ml conic tube with avoidance of the large chunks and debris. The cell suspension was centrifuged at 15000 rpm and the pellet was resuspended and seed at a density of ~2-2.5 million/ per 100mm dish. The media was changed in 24 hours. The cells were then maintained in Dulbecco’s Modified Eagle’s Medium (DMEM) supplemented with 10% FBS and 1% penicillin/streptomycin. The medium was refreshed every 4-5 days and passaged at 90%-100% confluency. The astrocyte cultures at passage 0-2 were cultured in 100 mm2 dishes and split into 60mm dishes or 35 mm dishes for experiments. Transfection of the GAT-1 mutants in astrocytes were carried out with PEI in astrocytes at passage 2. The total cDNAs is 9 µg for 100mm dish, 3µg for 60mm dish and 1 µg for 35mm dish at a ratio of 1µg:2.5µl PEI. The cells were harvest for experiments after 48 hrs.

**Radioactive ^3^H-labeled GABA uptake assay**

We followed our standard lab protocol as previously described^6,7^. Briefly, cells were cultured in 5 mm^2^ dishes 3 days before the GABA uptake experiment in DMEM with 10% fetal bovine serum and 1% penicillin/streptomycin. The cells were then transfected with equal amounts of the wildtype or the mutant GAT-1 cDNAs (1 µg) for each condition at 24 hrs or 48 hrs after plating. GABA uptake assay was carried out 48 hrs after transfection. The cells were incubated with preincubation solution for 15 min and then incubated with preincubation solution containing 1µci/ml ^3^H GABA and 10 µM unlabeled GABA for 30 min at room temperature. After washing, the cells were lysed with 0.25 N NaOH for 1 hr. Acetic acid glacial was added and lysates were then determined on a liquid scintillator with QuantaSmart. The flux of GABA (pmol/µg/min) was averaged with at least triplets for each condition at each transfection. The average counting was taken as n = 1. The untransfected condition was taken as a baseline that was subtracted from both the wildtype and the mutant conditions. The pmol/µg/min in the mutant was then normalized to the wildtype from each experiment, which was arbitrarily taken as 100%. The protocols for GABA reuptake assay in cultured mouse astrocytes, neuron or iPSC derived cells were modified from the GABA uptake protocol on HEK 293T cells. GAT-1 inhibitors CL-966 and NNC-711 were applied each time to make sure the radioactive counts were specific.

**Measurement of surface and total expression of GAT-1 using flow cytometry**

The protocol used for measurement of surface and total expression of GAT-1 using flow cytometry has been described previously for studies on GABA_A_ receptor mutations ^4,5^. Briefly, HEK293T cells were transfected using PEI reagent (40 kD, Polysciences) at a DNA: transfection reagent ration of 1:2.5 and harvested 48 hours after transfection. To express wild-type (GAT-1) and mutant GAT-1, a total of 3 µg of subunit cDNAs was transfected into 60mm^2^ dishes. The transfected HEK293T cells were removed from the dishes by trypsinization and then re-suspended in FACS buffer (phosphate buffered saline (PBS) supplemented with 2% FBS and 0.05% sodium azide). Following washes with FACS buffer and permeabilization with Cytofix/cytoperm (BD Biosciences, CA) for 15 minutes, cells were incubated with rabbit polyclonal anti-GAT-1 antibody (1:200) (Synaptic System, catalog no. 274 102 ) for 2 h. Cells were then washed with FACS buffer and then incubated with fluorophore Alexa-555or Alexa 488 conjugated goat anti-rabbit secondary antibody (1:400) for 1 h at 4°C. Cells were then washed with FACS buffer and the cell surface fluorophore intensity was determined using a 3-laser LSR II machine at Vanderbilt Flow Cytometry Core. The acquired data was analyzed using FlowJo 7.1 (Tree Star, Inc., OR).

**Confocal microscopy and image acquisition**

Live cell confocal microscopy was performed using an inverted Zeiss laser scanning microscope (Model 510) with a 63 × 1.4 NA oil immersion lens, 2-2.5 × zoom, and multi-track excitation^8^ . Cells were plated on poly-I-ornithine and Laminin-coated coverslips or glass-bottom imaging dishes at the density of 1-2 × 10^5^ cells per dish and co-transfected with 0.5 µg of the wildtype or the mutant GAT-1 plasmids with 0.5 µg of the ER marker ER^CFP^ per 35-mm glass-bottomed culture dish with PEI, as per our standard lab protocol. All images were obtained from live cells with single confocal sections averaged from 8 times to reduce noise.

**Immunohistochemistry related quantifications**

For all GAT-1 fluorescent signal, raw values of the protein intensity in the somatic region and neuronal process were measured in ImageJ and Metamorph. Randomly chosen non-overlapping fields were measured each time. All the cells were included in each field. The mean value was taken. If the staining was with fluorophore-conjugated secondary antibodies, the nuclear region of the same cells marked by TO-PRO-3 was used as background because the boundary of the nuclei was relatively clear. The protein intensity value of the somatic region for each cell was equal to the value of the somatic region subtracting the background value.

**Synchronized video-monitoring EEG recordings**

Surgery to implant the EEG head mount and the video-monitoring synchronized EEG recordings was conducted. Synchronized video EEGs were recorded from ~ 2 months old C57BL/6J mice one week after electrode implantation with synchronized EEG monitoring system from Pinnacle Technology. Briefly, mice were anesthetized with 1–3% isoflurane and four epidural electrodes (stainless steel screws affixed to one head mount) were placed on the brain surface, above the bregma, and secured in place with dental cement. EMG leads were inserted into the trapezius muscle. Mice were allowed to recover from the EEG head mount implantation surgery for 5-7 days before EEG recording. Ketofen (5mg/kg) was administered 5 min before surgery and for three-days post-surgery as an analgesic. Video-EEG monitoring lasted for 48 hrs. and mice were freely moving during EEG recordings. During EEG recordings, mice were checked daily for health concerns, and food/water access. The Racine Scale was used to identify mouse behaviors such as behavioral arrest, myoclonic jerks, or generalized tonic clonic seizures from the EEG recordings to determine if mice exhibit behavioral seizures**.** At least 48 hours of baseline EEG recordings were obtained and analyzed for each mouse.

**Reference List**

1. AM M, S K, JA T*, et al*. Directed differentiation and functional maturation of cortical interneurons from human embryonic stem cells. *Cell stem cell*. 05/02/2013 2013;12(5)doi:10.1016/j.stem.2013.04.008

2. Mermer F, Poliquin S, Rigsby K*, et al*. Common molecular mechanisms of SLC6A1 variant-mediated neurodevelopmental disorders in astrocytes and neurons. *Brain*. May 24 2021;doi:10.1093/brain/awab207

3. Romero-Morales AI, Robertson GL, Rastogi A*, et al*. Human iPSC-derived cerebral organoids model features of Leigh Syndrome and reveal abnormal corticogenesis. 2021-06-17 2021;doi:10.1101/2020.04.21.054361

4. Kang JQ, Shen W, Macdonald RL. Two molecular pathways (NMD and ERAD) contribute to a genetic epilepsy associated with the GABA(A) receptor GABRA1 PTC mutation, 975delC, S326fs328X. *J Neurosci*. Mar 04 2009;29(9):2833-44. doi:10.1523/JNEUROSCI.4512-08.2009

5. JQ K, W S, M L, MJ G, RL M. Slow degradation and aggregation in vitro of mutant GABAA receptor gamma2(Q351X) subunits associated with epilepsy. *The Journal of neuroscience : the official journal of the Society for Neuroscience*. 10/13/2010 2010;30(41)doi:10.1523/JNEUROSCI.2320-10.2010

6. K C, J W, J E*, et al*. A missense mutation in SLC6A1 associated with Lennox-Gastaut syndrome impairs GABA transporter 1 protein trafficking and function. *Experimental neurology*. 2019 Oct 2019;320doi:10.1016/j.expneurol.2019.112973

7. J W, S P, F M*, et al*. Endoplasmic reticulum retention and degradation of a mutation in SLC6A1 associated with epilepsy and autism. *Molecular brain*. 05/12/2020 2020;13(1)doi:10.1186/s13041-020-00612-6
